# Identification and Characterization of the Larval Settlement Pheromone Protein Components in Adult Shells of *Crassostrea gigas*: A Novel Function of Shell Matrix Proteins

**DOI:** 10.3390/ijms23179816

**Published:** 2022-08-29

**Authors:** Mary Grace Sedanza, Asami Yoshida, Hee-Jin Kim, Kenichi Yamaguchi, Kiyoshi Osatomi, Cyril Glenn Satuito

**Affiliations:** 1 Graduate School of Fisheries and Environmental Sciences, Nagasaki University, Nagasaki 852-8521, Japan; 2 Institute of Aquaculture, College of Fisheries and Ocean Sciences, University of the Philippines Visayas, Miagao, Iloilo 5023, Philippines; 3 Organization for Marine Science and Technology, Nagasaki University, Nagasaki 852-8521, Japan

**Keywords:** oyster larval ecology, conspecific cues, settlement induction, marine chemical ecology, biomineralization, post-translational modifications, lectin-glycan interaction

## Abstract

The global decline of natural oyster populations emphasizes the need to improve our understanding of their biology. Understanding the role of chemical cues from conspecifics on how oysters occupy appropriate substrata is crucial to learning about their evolution, population dynamics, and chemical communication. Here, a novel role of a macromolecular assembly of shell matrix proteins which act as *Crassostrea gigas* Settlement Pheromone Protein Components in adult shells is demonstrated as the biological cue responsible for gregarious settlement on conspecifics. A bioassay-guided fractionation approach aided by biochemical and molecular analyses reveals that Gigasin-6 isoform X1 and/or X2 isolated from adult shells is the major inducing cue for larval settlement and may also play a role in postlarva–larva settlement interactions. Other isolated Stains-all-stainable acidic proteins may function as a co-factor and a scaffold/structural framework for other matrix proteins to anchor within this assembly and provide protection. Notably, conspecific cue-mediated larval settlement induction in *C. gigas* presents a complex system that requires an interplay of different glycans, disulfide bonds, amino acid groups, and phosphorylation crosstalk for recognition. These results may find application in the development of oyster aquacultures which could help recover declining marine species and as targets of anti-fouling agents.

## 1. Introduction

The Pacific oyster *Crassostrea gigas* (Thunberg, 1973) is a benthic bivalve mollusk that has been introduced worldwide and has become one of the most well-studied organisms due to its biological, ecological, and aquacultural importance [1,2]. Pacific oysters, like other oyster species, act as foundation species by creating habitats for other estuarine species and providing key ecosystem services to human communities around the world [3,4,5]. Pacific oysters have been widely used as a model species for studying molecular genetics [1,2,6], developmental biology [7,8,9,10], biomineralization [11,12], and adaptation to coastal environments under climate change [13]. Beck et al. [14] have reported that oyster populations have significantly declined on a global scale, with an estimated 85% of oyster reefs gone, far outnumbering the projected loss of coral reefs. Understanding how oysters such as *C. gigas* occupy appropriate substrata is crucial to learning about their evolution, population dynamics, and chemical communication.

Population dynamics are controlled by recruitment and post-settlement survival in marine invertebrates such as oysters. Finding a suitable settlement substrate is crucial for their survival, growth, and reproduction [15,16,17]. Oysters are also known to form reef-like structures characterized by monospecific aggregations. The benefits of such gregarious settlement behavior seem to prove beneficial in most sessile invertebrate species as this facilitates increased post-metamorphic survival [18,19] and synchronized fertilization [19,20]. Oyster substrate selection behavior is influenced by a variety of chemical cues that may come from bacterial biofilms, prey or predator, conspecifics, and macroalgal hosts [15,21].

Chemical signaling by a wide array of water-soluble or surface-bound cues such as pheromones associated with conspecific adults has been reported to influence gregarious behavior in oysters and induce larval settlement. Cole and Knight-Jones (1939) were the first to report on gregarious behavior in oysters specifically for *Ostrea edulis,* and their work was later confirmed by the study of Bayne [22]. Bayne demonstrated that larval settlement could be induced by the application of *O. edulis* tissue extracts to a surface [22]. This eventually led to more studies investigating the effect of chemical cues from conspecifics and their involvement in gregarious behavior for other oyster species. Such studies include shells in *C. gigas* [23,24,25,26], *C. virginica* [27,28], *C. ariakensis* [29]; soft tissue homogenates in *Ostrea edulis* [22]; water pre-conditioned by adults of *C. ariakensis* [29], *C. gigas* [30,31,32], *C. virginica* [33,34,35], and *O. puelchana* [36]; oyster shell liquor or extrapallial fluid of *C. virginica* [37]; and ammonia in *C. gigas* [38]. The chemical basis for such gregarious settlement behavior of oysters is not yet fully clarified and the identification and characterization of the active cues that drive such induction of the conspecific cue-mediated larval settlement phenomenon have not yet been achieved. In this study, settlement is defined as the sequential transition of a competent pediveliger larva to a postlarva, which includes the attachment to a substrate and then eventual metamorphosis.

Diederich (2005) demonstrated through field experiments that larval recruitment was higher on its conspecifics, whether on *C. gigas* as a living substrate or on its dead shells [25]. In addition, Vasquez et al. [23] have reported an unidentified 55 kDa HCl-soluble organic matrix extract from *C. gigas* shells that may mediate larval settlement induction among its conspecifics, and which could only be visualized under the Stains-all staining method. They have also demonstrated in another study the involvement of a WGA-binding sugar chain from the shell extract that could mediate conspecific cue-settlement induction [24]. Previous studies, therefore, tried to pursue the investigation of the nature and properties of this chemical cue by using a different chemical solution that has been widely used in extracting molluscan shell matrix proteins, i.e., Ethylenediaminetetraacetic acid (EDTA) [39,40]. A previous study has demonstrated that a chemical cue from adult shells (*C. gigas* EDTA-soluble shell extract, CgSE) was isolated that could induce larval settlement in a positive-concentration-dependent manner and that GlcNAc and Sialic acid sugars from the chemical cue were found to interact with a WGA lectin-like receptor from the oyster larva [26].

Shells of *C. gigas* are made up of 99% calcium carbonate and around 0.5% occluded organic matrix [11]. This organic matrix is composed of a combination of proteins, glycoproteins, and polysaccharides that self-assemble and govern the shell’s calcium carbonate polymorph (calcite, aragonite), crystallite size, shape, and texture [11]. Several molluscan shell matrix proteins (SMPs) have been recently identified in shells, including that of *C. gigas*, but much of the focus has been on their biomineralization roles [41,42]. One of the main challenges to studying SMPs is their inherent difficulty to isolate, characterize and obtain enough of an amount for functional analyses due to their close association with the mineral phase, highly charged nature, and presence of post-translational modifications (PTMs) [43,44]. To overcome this, a larval settlement-guided approach was developed to locate the signal molecule from *C. gigas* conspecific shells, aided by various biochemical analyses using different gel staining methods and enzyme treatments, mass spectrometry analyses, bioinformatics analyses of protein sequences, and gene expression analysis of a selected gene between the *C. gigas* pediveliger and postlarvae. Previously identified chemical cues in another marine invertebrate species, i.e., barnacle, *Balanus amphitrite,* were shown to be induced by some polypeptides processed from a single precursor protein encoded in a single gene, which was designated to be the Settlement-Inducing Protein Complex (SIPC) [45,46]. However, a novel role of a macromolecular assembly of matrix proteins that act as an aggregating settlement cue is demonstrated for the first time in this study and hereon named *Crassostrea gigas* Settlement Pheromone Protein Components (CGSPPC) that cooperatively function by at least two or more unrelated gene products. This study elucidates the identity, characteristics, and mechanisms underlying the conspecific cue-mediated induction of *C. gigas* larval settlement. A discussion is made on the properties and contribution of each protein component to settlement induction, the role of PTMs, and lastly, the molecular basis of the conspecific cue-mediated larval settlement mechanism.

## 2. Results

### 2.1. Protein Separation and Identification of the Settlement Inducing Cues from Crassostrea gigas EDTA-Soluble Shell Matrix Proteins

#### 2.1.1. Protein Separation

To locate the signal molecule from the *C. gigas* adult shell EDTA-soluble extract (CgSE), two bioassay-guided protein separation approaches were performed. In this first approach (Supplementary text A, Supplementary Figures S1–S4), the identification and characterization of the protein extract that could only be visualized by the Stains-all staining method reported in Vasquez et al. [23,24] were further pursued.

**Figure 1:**
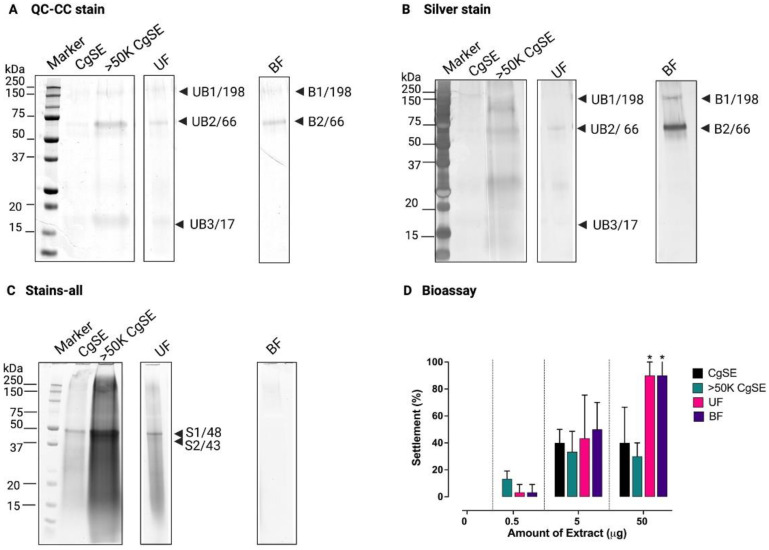
SDS-PAGE analyses and larval settlement responses to *Crassostrea gigas* EDTA-soluble shell matrix proteins and its fractions after elution from WGA lectin affinity chromatography. (**A**–**C**) SDS-PAGE profile of fractions eluted from WGA lectin affinity chromatography under different staining methods. (**D**) Settlement percentages of *C. gigas* larvae response with varying amounts of different forms of CgSE and fractions eluted after WGA affinity chromatography. All larvae were exposed to the settlement-inducing cue for 24 h. Asterisks “*” indicate significant differences among varied forms of CgSE determined via quasi-binomial glm (*p* < 0.05). Missing bars in the figure indicate no settlement. Data are the means (SD) of three replicates. Abbreviations: UF, Unbound fraction; BF, Bound fraction; UB1-UB3, B1, B2, S1, and S2 bands correspond to the identified proteins in the mass spectrometry analyses and are summarized in Table 1. This figure was created using BioRender.com (accessed on 25 July 2022) and GraphPad 9 software program.

To determine whether there could be other settlement-inducing factors in CgSE, the crude form of CgSE was subjected to another round of protein separation by ultrafiltration, but this time separating it directly into <50K and >50K fractions. The resultant >50K CgSE fraction may include high molecular weight settlement factors that might have been previously missed. To examine whether this >50K CgSE obtained from the second approach could bind to WGA lectin, a WGA-agarose-bound lectin affinity chromatography was performed (Figure 1 and Supplemental Figure S5). By using QC-CC and Silver staining methods, 198 (UB1), 66 (UB2), and 17 (UB3) kDa bands were detected in the unbound fraction (UF) component, while the 198 (B1) and 66 (B2) kDa bands were found in the bound fraction (BF) component (Figure 1A,B). On the other hand, under the Stains-all staining method, 48 (S1) and 43 (S2) kDa dominant bands were detected in the UF component (Figure 1C).

In Figure 1D, no settlement response was observed in multi-wells in the absence of settlement-inducing cues, i.e., CgSE, UF, and BF components. Notably, both the UF and BF components showed similar concentration-dependent settlement-inducing patterns in all amounts of extract exposure and a 90% settlement response at 50 µg (*p* < 0.05). This result indicates that the signaling molecule was found in the BF component that may have GlcNAc and Sialic acid-containing sugar moieties which could bind with WGA lectin and have been hypothesized to mediate settlement in *C. gigas* [26]. On the other hand, although the protein components in the unbound fraction may not have bound to the WGA lectin, it indicates that it could still induce settlement. No mortality was recorded in all bioassay experiments.

#### 2.1.2. Identification by Mass Spectrometry

To identify the dominant matrix proteins of CgSE, both the UF and BF components were subjected to Cys-β-propionamidation prior to SDS-PAGE and further analyzed by peptide mass fingerprinting (PMF) following in-gel tryptic digestion. As summarized in Table 1 and shown in Figure 1, three protein groups were identified. Annotated MS spectra are provided in Supplemental Table S1. Availability of the gene sequences was evaluated with *C. gigas* CDS and NCBInr databases. The disparity of the molecular masses between the observed and theoretical mass may be due to the additional presence of post-translational modifications [44].

The major band UB2 (66 kDa) in the unbound fraction (UF) and band B2 in the WGA column-bound fraction (BF) were identified as Gigasin-6 isoform X1 and/or X2. The minor band UB1 (198 kDa) in the unbound fraction (UF) and band B1 in the WGA column-bound fraction (BF) band were also identified as the same Gigasin-6 isoform(s). This 198 kDa band could be an SDS-resistant trimer of Gigasin-6 (66 kDa) as reported in certain other proteins [47,48,49]. The 17 kDa band (UB3) was identified as (putative) mature proteins from two gene product(s) of Surface protein P12p-like that were indistinguishable from each other. Furthermore, both the QC-CC and silver stain profiles of Gigasin-6 isoform X1 and/or X2 and its trimer showed that a more intense band pattern of this protein was visualized in the bound fraction component than in the unbound fraction, indicating that the majority of this protein bound to the WGA lectin. The low amount of Gigasin-6 isoform X1 and/or X2 and its trimer that remained in the unbound fraction may be due to the lack of available binding sites on the immobilized WGA lectin resin or as a result of the oversaturated presence of the same protein. Those that could not bind to the lectin might have flowed through together with other protein components in the unbound fraction. On the other hand, the 43 and 48 kDa bands which could only be visualized with Stains-all staining were not successfully identified due to a lack of tryptic cleavage sites and also due to stain interference [44].

### 2.2. Biochemical and Molecular Characterization of the Settlement-Inducing Cues from C. gigas EDTA-Soluble Shell Matrix Proteins

#### 2.2.1. Biochemical Characterization

##### Post-Translational Modifications (PTMs)

**Figure 2:**
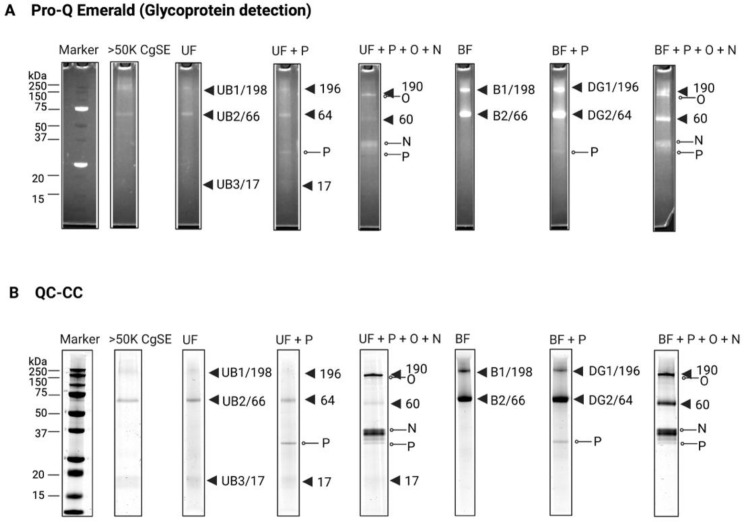
Enzymatic deglycosylation and glycoprotein staining profiles. (**A**) Unbound and Bound fractions eluted from WGA affinity chromatography were enzymatically deglycosylated with PNGase F (P), *O*-glycosidase (O), and Neuraminidase (N). Ten µg of each sample was loaded on a 12% SDS-PAGE and the gel was stained by Pro-Q emerald for Glycoprotein detection. (**B**) The gel was subsequently double-stained with QC-Colloidal Coomassie (QC-CC) Stain. Abbreviations: UF, Unbound Fraction; BF, Bound Fraction; UB1-UB3, B1, B2, DG1, DG2, S1, and S2 bands correspond to the identified proteins in the mass spectrometry analyses and are summarized in Table 1 and Table 2. This figure was created using BioRender.com (accessed on 25 July 2022).

**Figure 3:**
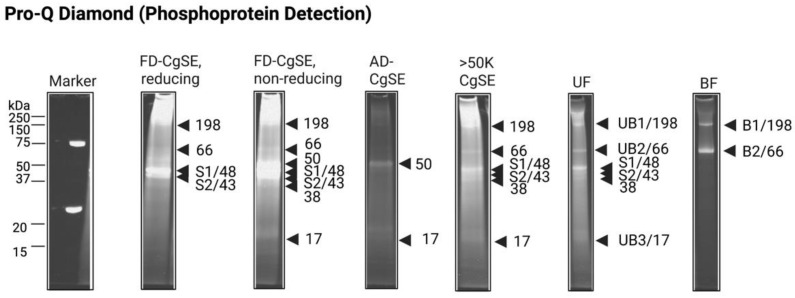
Phosphoprotein detection. Ten µg of the sample was loaded on each lane and stained with Pro-Q Diamond for phosphoproteins. Abbreviations: FD, Freeze-dried CgSE, AD, Air-dried CgSE; UF, Unbound Fraction; BF, Bound Fraction; UB1-UB3, B1, B2, S1, and S2 bands correspond to the identified proteins in the mass spectrometry analyses and are summarized in Table 1. This figure was created using BioRender.com (accessed on 25 July 2022).

To examine the presence of PTMs in the WGA affinity chromatography-eluted UF and BF components, glycoprotein and phosphoprotein detection were performed as shown in Figure 2 and Figure 3, respectively. Bands that showed fluorescence staining were confirmed according to each staining method. The mass spectrometry analyses of the identified bands are shown in Table 1 and Table 2.

Enzymatic deglycosylation and Pro-Q Emerald staining revealed that Gigasin-6 isoform X1 and/or X2 (66 kDa) and its trimer (198 kDa) is a highly glycosylated protein (Figure 2). Gigasin-6 isoform X1 and/or X2 (66 kDa) and its trimer (198 kDa) found in the unbound fraction (UF) component showed a weak-band fluorescence compared to its bound fraction (BF) component. After PNGase F treatment alone, the Gigasin-6 isoform X1 and/or X2 trimer (198 kDa) showed a slight band shift from 198 (UB1/B1) to 196 (DG1) kDa while, in the native form of this protein (66 kDa), a slight band shift from 66 (UB2/B2) to 64 kDa (DG2) was also observed on both the UF and BF components. Simultaneous removal of *N*-glycans and *O*-glycans on the polypeptide backbone [50] in both the UF and BF components showed a significant band shift for Gigasin-6 isoform X1 and/or X2 and its trimer. As a result, the 198 kDa band trimer shifted to a 190 kDa band, while the native form of Gigasin-6 kDa (66 kDa) shifted to 60 kDa. The fluorescence intensity pattern within each of the UF and BF components of the deglycosylated Gigasin-6 isoform X1 and/or X2 and its trimer became weak compared to their glycosylated forms. These results demonstrate that Gigasin-6 isoform X1 and/or X2 and its trimer contained several types of *N*-glycans containing GlcNAc and Sialic acid residues (for PNGase F treatment) [23,51]; *O*-glycans containing non-reducing terminals of *N*-acetylgalactosamine Core 1 and Core 3 *O*-linked disaccharides (for *O*-glycosidase treatment) [50], and α2-3-, α2-6-, and α2-8-linked *N*-acetyl-neuraminic acid residues (for Neuraminidase treatment) [50]. Notably, both the 190 kDa and 60 kDa deglycosylated bands of Gigasin-6 isoform X1 and/or X2 continued to show a positive fluorescence stain, albeit a weaker one, after the sequential treatment of a mixture of deglycosylation enzymes. This suggests that Gigasin-6 isoform X1 and/or X2 may possess other types of uncleaved *O*-linked glycans. A similar finding was also reported by Vasquez et al. [23] regarding the presence of GlcNAc residues on the settlement-inducing cue. On the other hand, Surface Protein P12p-like was shown to have a faint fluorescence band pattern in the UF component (Figure 2A) indicating that it is lightly glycosylated, whereas the 43 and 48 kDa Stains-all-stainable acidic protein bands showed no Pro-Q Emerald fluorescence. This may indicate that the Stains-all-stainable acidic protein bands may have no or few glycosylation sites. In addition, no band shift was observed on these protein bands after enzymatic deglycosylation treatments.

To confirm the identity of Gigasin-6 isoform X1 and/or X2 (66 kDa) and its trimer (198 kDa in the WGA column-bound fraction (DG 1 and 2 bands) after treatment with PNGase F, mass spectrometry analysis was performed. The WGA column-bound fraction (BF) proteins were subjected to Cys-β-propionamidation prior to SDS-PAGE and further analyzed by MS/MS ion search following in-gel tryptic digestion. Annotated MS/MS spectra are provided in Supplemental Table S2. These same MS spectra were used to further determine the *N*-glycosylation sites of Gigasin-6 isoform X1 and/or X2 in the WGA column-bound fraction (BF) after treatment with PNGase F.

For the phosphoprotein detection, a separate set of samples from the UF and BF components together with other forms of CgSE were subjected to SDS-PAGE and visualized by the Pro-Q Diamond staining method as shown in Figure 3. The results revealed a high fluorescence intensity on the 43 (S2), 48 (S1), and 50 kDa Stains-all-stainable acidic bands as well as Gigasin-6 isoform X1 and/or X2 (66 kDa) and its trimer (198 kDa). This indicates that these bands were highly phosphorylated. However, the 38 kDa Stains-all-stainable acidic protein band and Surface protein P12p-like (UB3/17 kDa) showed fluorescence but with a weaker signal compared to other stained proteins and may contain few phosphorylated sites. This finding suggests that all these dominant proteins in CgSE were phosphorylated.

##### Characterization of Stains-All-Stainable Acidic Proteins by Protease and Chemical Deglycosylation Treatments

**Figure 4:**
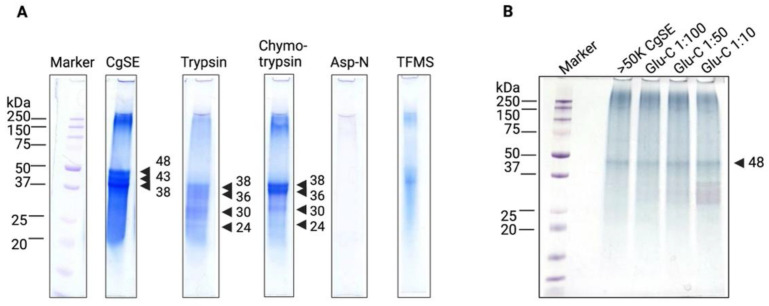
SDS-PAGE profiles of CgSE and >50K CgSE-protease and chemically deglycosylated treated samples. (**A**) Forty µg of CgSE was treated with various endoproteinase enzymes and stained with Stains-all. (**B**) Ten µg of >50K CgSE was treated with Glu-C Endoproteinase under different enzyme-to-protein ratios and stained with Stains-all. Abbreviation: CgSE, *Crassostrea gigas* shell EDTA extract; TFMS, Trifluoromethanesulfonic acid. This figure was created using BioRender.com (accessed on 25 July 2022).

To further determine the characteristics of the protein bands that were consistently visualized by the Stains-all staining method, CgSE was treated separately with Trypsin, Chymotrypsin, Asp-N, and Trifluoromethanesulfonic acid (TFMS) for chemical deglycosylation. Figure 4A shows that without any enzyme treatment, CgSE contained 38, 43, and 48 kDa bands. After enzyme treatment, a 10 kDa band shift was observed on CgSE treated with trypsin and chymotrypsin. Notably, the band shift in both treatments showed similar patterns wherein 24, 30, 36, and 38 kDa newly appeared. However, these target bands were not completely cleaved and showed some resistance to these enzymes. The cleavage specificity of trypsin is on the carboxyl side of arginine and lysine residues while chymotrypsin acts on the carboxyl side of tyrosine, phenylalanine, tryptophan, and leucine residues. Interestingly, after Asp-N treatment, all bands disappeared owing to its fragmentation into very low molecular weight sizes undetectable under SDS-PAGE. Asp-N specifically cleaves the amino side of aspartic acid residues. This indicates that CgSE, which is predominantly composed of Stains-all-stainable acidic proteins, was rich in aspartic acid. This is typical for proteins found in shells [40,52] and which may exhibit properties common to intrinsically disordered proteins [52]. Moreover, TFMS-treated CgSE showed no distinguishable bands; instead, a diffused and scaling smeared pattern from 20 kDa to >250 kDa appeared. Chemical deglycosylation with TFMS allows the removal of monosaccharides without damaging the peptide [53]. However, in this experiment, the results indicate that peptide bond hydrolysis may have occurred, thereby creating such a band pattern.

**Figure 5:**
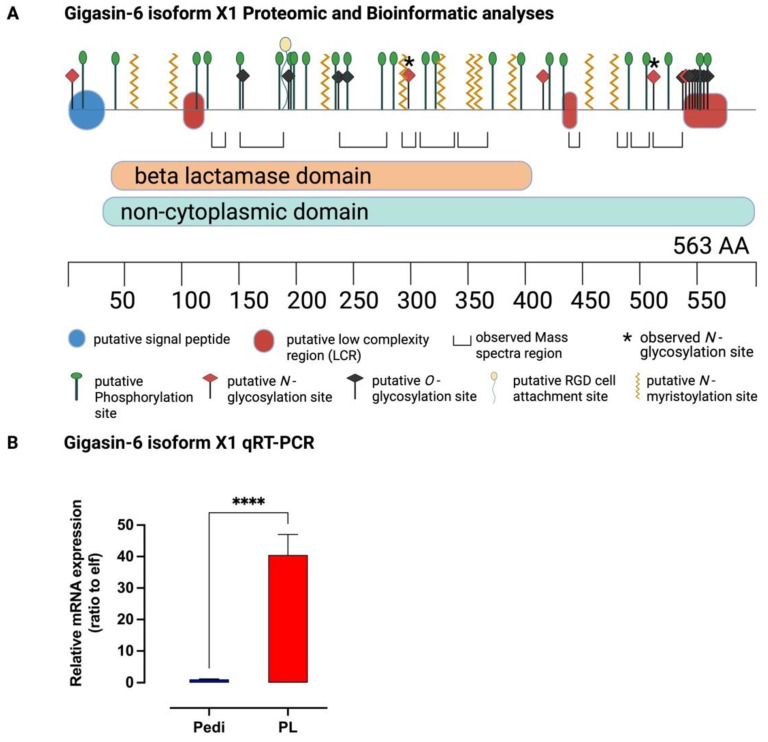
Molecular characterization of Gigasin-6 isoform X1. (**A**) Graphical representation showing its protein length, signal peptide, putative domains, motif, and post-translational modifications identified by mass spectrometry and bioinformatic analyses. The putative phosphorylation sites (24 out of 70) in this model highlight those which may be involved in signal-transduction processes. The observed region indicates the location of the mass spectra identified by mass spectrometry analysis. Figure 5A was created using BioRender.com, accessed on 25 July 2022. (**B**) Relative mRNA expression levels of Gigasin-6 isoform X1 gene before (Pediveliger, Pedi) and after (postlarvae, PL) settlement induction by the CgSE using quantitative real-time PCR. Data are expressed as the mean (SD) (*n* = 3). Values with “****” are significantly different (*p* < 0.0001). Figure 5B was created using GraphPad 9 software program.

To examine the presence of glutamic acid residues in the Stains-all-stainable acidic proteins, >50K CgSE was treated with an endoproteinase, Glu-C, at different enzyme-to-protein ratios. Figure 5B consistently showed an uncleaved prominent 48 kDa band after varied Glu-C concentrations. This indicates that this matrix protein was Glu-C resistant and may be due in part to the presence of Glu-C proteinase-resistant Glu-X bonds, such as Glu-Asp, Glu-Asn, and Glu-Gly, where X is linked to Pro or Gln residues which could yield a missed cleavage [54].

In addition, in the first protein separation approach, the Stains-all-stainable acidic proteins showed that under reducing conditions, no settlement-inducing activity was detected from the isolated polypeptides in the active fraction F2, i.e., with the addition of 2-mercaptoethanol (Supplementary text A.3, Figure S4). Conversely, under non-reducing conditions, significant settlement-inducing activities were observed at the 35, 38, and 48 kDa polypeptide bands (Supplementary text A.3, Figure S4). This suggests that disulfide bonds might be necessary for the folding and stabilization of the settlement-inducing factors in this cue [44].

The overall SDS-PAGE profiles in Figure 4 suggest that aspartic acid-rich proteins were the major components of the EDTA-soluble organic matrix in this study as is reported in other bivalves [40,55]. This implies that these proteins may also bind to calcium [55] and that they are intrinsically disordered proteins. Moreover, as the amount of extract loaded on each lane increased, the intensity of the smeared background along each lane and the appearance of aggregated proteins close to the loading lane was noticeable. This also confirms the presence of a high level of post-translational modifications found in this extract component [39,56].

#### 2.2.2. Molecular Characterization

To examine further the characteristics of the dominant protein bands in the UF and BF components following WGA affinity, chromatography, bioinformatic characterization of all protein sequences, mass spectrometry, and quantitative real time-PCR (qRT-PCR) (for Gigasin-6 isoform X1 only) analyses were performed.

##### Gigasin-6 isoform X1 and/or X2

Peptide Mass Fingerprinting analysis revealed that the genes encoding both 66 kDa and 198 kDa bands were identified as Gigasin-6 isoform X1 and/or X2 (Table 1). Mass spectrometry profiles of this gene showed identical mass-to-charge (m/z) ratio signals that made it difficult to differentiate both isoforms (Supplemental Table S1). Multiple sequence alignment of the precursor Gigasin-6 and its two isoforms confirmed that these isoforms contained identical sets of amino acid sequences except that Gigasin-6 isoform X1 possessed an additional nine residues (DSNNPDSTL) at positions 115 to 123 (Supplemental Figure S6).

A graphical representation of the predicted and experimentally observed properties of Gigasin-6 isoform X1 is shown in Figure 5 A. Bioinformatic analyses on the Gigasin-6 isoform X1 protein sequence suggest five *N*-glycan, twelve *O*-glycan, and seventy putative phosphorylation sites; one putative RGD cell attachment sequence at positions 177-179; and ten putative *N*-myristoylation sites (Figure 5A, Supplementary text B1, Supplemental Figure S6). If the *N*-myristoylation sites are functional, each site may determine the final location of this protein on the shell layer [55]. Multiple sequence alignment of Gigasin-6 isoform X1 showed some amino acid residues, sequence insertions, and deletions that were unique to themselves in comparison with other bivalves (Supplementary Figure S7). The identification of Gigasin-6 isoform X1 and/or X2 in the adult shells of *C. gigas* is consistent with a previous study by Mouchi et al. [57]. Mouchi et al. [57] also reported that this protein could interact synergistically with other acetic acid-soluble matrix proteins to regulate in vitro the formation of calcium carbonate through induction of polycrystalline aggregates.

To identify the actual *N*-glycosylation sites in this protein, the peptide mass fingerprint patterns of the glycosylated and deglycosylated bands B5 and B7, respectively, were compared [58] (Table 1 and Table 2, Figure 2, Supplementary text B2, Figure S8). The results revealed four *N*-glycosylation site-related m/z signals corresponding to two *N*-glycosylation peptide sequences (^521^**NST**YIEAFTVDKFDDAKFER^540^ and **^291^**FMNYLLG**NGS**IPGTNDVLLAK^309^). This study also reports for the first time an unrecorded actual *N*-glycosylation site-related m/z 2525.21 signal from this protein. This result highlights the importance of combining biochemical, bioinformatic, and mass spectrometry techniques to explore and verify undiscovered glycan moieties that may play crucial roles in biological processes [58].

As shown in Figure 5B, the results of the qRT-PCR analysis showed that Gigasin-6 isoform X1 was highly expressed in the CgSE-induced postlarvae than in the pediveliger larvae (*p* < 0.001). This finding further supports the transcriptome data in a previous study where Gigasin-6 isoform X1 was differentially expressed and highly upregulated in the CgSE-induced postlarvae [59]. In addition, this result is consistent with the findings of Xu and Zhang [6] where the Gigasin-6 gene was significantly upregulated in the epinephrine-induced postlarvae of *C. gigas*. It is often observed that in a large-scale culture of oyster larvae, once a batch of postlarvae begins to settle on the culture tank, other competent pediveliger larvae are also induced to settle. In this present study, the isolated Gigasin-6 isoform X1 and/or X2 found in the adult shells that could induce larval settlement was also highly expressed in the CgSE-induced postlarvae. This indicates that it may function in postlarva–larva settlement interactions such that in the presence of settled juveniles, other non-settled larvae may also be induced to settle.

##### Stains-All-Stainable Acidic Proteins

The close association of the Stains-all-stainable acidic proteins with the biomineral phase and their highly acidic nature made it difficult to confirm their identity. The in-gel and in-solution protease digests (Figure 1 and Figure 4) of all the Stains-all-stainable acidic protein components did not reveal any peptide with sequences corresponding to those found in the *C. gigas* NCBI database. Asp-N digests were too fragmented and did not yield any identifiable sequences. Although mass spectrometry analysis could not successfully identify the putative settlement inducer from the Stains-all-stainable acidic proteins due to their large-sized trypsin, chymotrypsin, and Glu-C digests, all bands showed similar chemical signatures, suggesting that these bands were fragments of proteins that may have similar chemical structures. Since a Stains-all-stainable shell matrix protein has been identified from other species; i.e., Folian cv1 (*C. virginica*) [40], the Folian cv1 homolog of *C. gigas* was searched for by BLAST and a gene-encoding Dentin sialophosphoprotein-like protein (CGDSP, XP_034310169.1, Supplementary text C, Figure S9) was identified. Prediction of intrinsically unstructured proteins and detection of low complexity regions (LCRs) were carried out using IUPred3 software. IUPred3 analysis of the Dentin sialophosphoprotein-like protein sequence revealed that it is considered to be a putative intrinsically disordered protein (Supplementary text C). In a previous study, the dentin sialophosphoprotein (CGDSP) gene was found to be highly upregulated in the CgSE-induced postlarvae transcriptome [59]. Nonetheless, in this study, the ability of these isolated Stains-all-stainable acidic proteins to induce larval settlement in both the first and second protein separation approaches was demonstrated to be consistent with the report of Vasquez et al. [23] where a 55 kDa Stains-all-stainable acidic protein from an HCl-soluble shell extract was found to induce conspecific settlement in *C. gigas* larvae.

##### Surface Protein P12p-like

Peptide Mass Fingerprinting of the 17 kDa band was identified as the product(s) of two isoform genes of Surface protein P12p-like (accession numbers: XP_034319257.1 and XP_034321529.1) as shown in Table 1. In Supplemental Figure S10A, a sequence alignment comparison of these two variant gene products indicates they had identical amino acid sequences except for one substitution of an amino acid residue at position seven, where a Phe was replaced by Leu, in a putative signal peptide. The protein sequence of this protein was predicted only by automated computational analysis derived from a genomic sequence in *C. gigas* [60]. Its functional role remains unexplored. This is the first report on the mass spectrometry and PTM characterization of this protein. The results in this study indicate that it was slightly glycosylated and phosphorylated. A closer look at its few putative *N*-and *O*-glycosylation and phosphorylation sites concur with the observed results in the SDS-PAGE analyses (Figure 2 and Figure 3). It also contained some putative *N*-myristoylation sites (Supplementary text D, Figure S10). To compare the *C. gigas* surface protein P12p-like (CGS12P) with its homologs, a multiple sequence alignment was performed. Notably, Supplemental Figure S11 shows this protein had variation and low homology with other proteins specific to *Crassostrea gigas*, but no homology was found in other organisms. Interestingly, this is the first report implicating the actual presence of this protein as part of the adult shell matrix in *C. gigas*.

The overall biochemical and molecular characterization of Gigasin-6 isoform X1 and/or X2, CGS12P, CGDSP, and other Stains-all-stainable acidic bands in this study suggest that their amino acid composition showed some amino acid residues, sequence insertions, and deletions that are unique to each of these proteins in *C. gigas* in comparison with other organisms, and that all these isolated proteins possessed post-translational modifications (glycosylation and phosphorylation) that may play crucial roles in cell signaling processes and strengthen recognition processes for conspecific cue-mediated larval settlement induction.

## 3. Discussion

This study reports a novel role of a macromolecular assembly of shell matrix proteins that cooperatively function as the *Crassostrea gigas* Settlement Pheromone Protein Components, which is demonstrated as the biological cue responsible for gregarious settlement on conspecifics.

In this present study, a bioassay-guided protein separation approach was conducted to locate the key molecule from conspecific adult shells responsible for gregarious settlement in *C. gigas*. The results showed that in the first protein separation approach, a group of Stains-all-stainable acidic proteins were isolated. The extracted proteins were capable of inducing settlement and possess characteristics inherent to proteins with intrinsically disordered regions (IDPs) [42,52,61,62]. This result is consistent with the report of Vasquez et al. [23] where a 55 kDa Stains-all-stainable acidic protein from an HCl-soluble shell extract was found to induce settlement in *C. gigas* larvae. In the second protein separation approach, although Gigasin-6 isoform X1 and/or X2, which is bound to a WGA lectin resin, could induce a high concentration-dependent settlement inducing activity, the unbound fraction could also elicit the same settlement-inducing pattern. This unbound fraction component contained a combination of a low amount of Gigasin-6 isoform X1 and/or X2, a moderate amount of CGS12P, and the dominant Stains-all-stainable acidic proteins including the 48 kDa band, putatively identified as CGDSP. One possible reason for such a settlement-inducing pattern in the unbound fraction component could be that only a small amount of Gigasin-6 isoform X1 and/or X2 might be needed to contribute to a settlement-inducing effect. However, the Stains-all-stainable acidic proteins which were shown in the first protein separation approach to induce settlement could also be contributing to the increased settlement signal in this fraction component. Moreover, whether CGS12P is a co-factor or not has to be investigated and confirmed by further study. These protein components in the unbound fraction that may not have bound to the WGA lectin indicate that it could still induce settlement by interacting with the oyster larvae via an unknown signaling pathway. Additionally, separating the dominant protein components individually showed lower settlement-inducing effects. The overall results of the bioassay-guided protein separation, biochemical, and molecular analyses suggest that these dominant EDTA-soluble shell matrix proteins may be synergistically interacting together to form a macromolecular assembly capable of creating a stable and strong aggregating signal for conspecific settlement which is hereon named the *Crassostrea gigas* Settlement Pheromone Protein Components (CGSPPC). However, whether or not CGSPPC forms a `complex’ or is dispersed on the shell has to be studied in the future.

One possible reason that may explain the synergistic settlement-inducing effect of the CGSPPC could be the hypothesis proposed by Pancsa et al. [61]. Pancsa et al. [61] proposed that intrinsically disordered region-containing proteins do not have a function on their own, but when these proteins form dynamic and non-stoichiometric supramolecular assemblages, novel and functional properties emerge [61]. The possible presence of the putative CGDSP and other IDPs acting as scaffold proteins may provide the structural framework on which other settlement-inducing proteins could anchor via phosphorylation interactions and induced proximity [63]. IDPs which are involved in biomineralization are frequently very extended and disordered [62]. However, their disordered structure is integral to how these proteins fulfill their function [62]. IDPs are also known as assemblers which could simultaneously bind to multiple ligands as macromolecular assemblies [61,62]. The meta-stable conformation of the Stains-all-stainable acidic proteins in this study, as an IDP, allows them to bind to their protein partners as well as act with high specificity and relatively low affinity [62]. The capacity of the Stains-all-stainable acidic proteins to interact with Gigasin-6 isoform X1 and/or X2 and CGS12P may have contributed to changing or adjusting the structures and functions of all multiple partners enabling them to collectively intensify the settlement-inducing signal [64]. This might be the underlying reason that explains the comparable high settlement-inducing activity of the unbound fraction component despite having most of the Gigasin-6 isoform X1 and/or X2 separated into the bound fraction component. Further investigation is needed to clarify the specific binding sites of these protein components within *C. gigas* adult shells. Notably, the Stains-all-stainable acidic proteins were found to be trypsin-, chymotrypsin-, and Glu-C-resistant indicating that this set of proteins was highly modified and had no or few cleavage sites for these enzymes. In addition, Vasquez et al. (2013) reported that the Stains-all-stainable acidic protein 55 kDa putative settlement cue from *C. gigas* HCl-soluble shell matrix extract was stable even at 200 °C [23]. This stability of some shell matrix proteins at high temperatures has also been demonstrated in other oyster shells [65]. This proves advantageous for oysters that have to protect themselves against enzyme secretions from shell-boring epibiont organisms, extreme desiccation exposure, and possible predation [42]. Furthermore, these properties of the Stains-all-stainable acidic proteins suggest the protective role they may confer on their interacting partners within the shell matrix, thus enabling this conspecific cue to elicit a strong and stable aggregating signal even under harsh environmental conditions. This is a point that may have contributed to their evolutionary success as benthic organisms.

**Figure 6:**
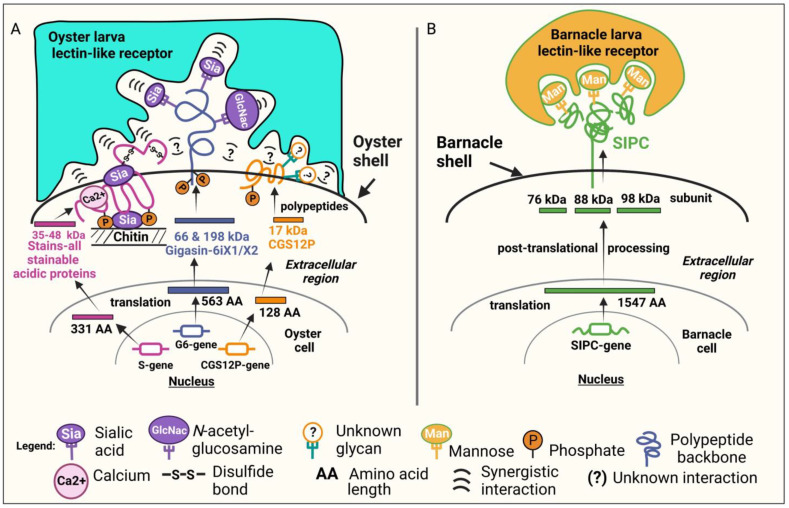
Comparison of settlement-inducing cues in oysters and barnacles. (**A**) Supramolecular interaction of several unrelated genes from adult oyster shells as *Crassostrea gigas* Settlement Pheromone Protein Components (CGSPPC). (**B**) Settlement-Inducing Protein Complex (SIPC) is encoded in a single gene in the barnacle, *Balanus amphitrite*. For comparison purposes, the authors illustrated the concept model in Figure 6B based on the reports by Dreanno et al. [45] and Matsumura et al. [46]. This figure was created using BioRender.com (accessed on 25 July 2022).

A graphical representation of CGSPPC and its comparison with barnacle SIPC is shown in Figure 6. The present findings showed that several signaling molecules (CGSPPC) from *C. gigas* conspecific adult shells could induce settlement while those reported in other marine invertebrates come from a single signaling molecule such as the α-macroglobulin-like gene that encodes the Settlement-Inducing Protein Complex (SIPC) in barnacles [45,46]. In Sedanza et al. [26] their results indicate that the induction of settlement in oyster larvae may not only require lectin–glycan interactions but also amino acids and other chemical groups [66,67] including phosphates present at the site of chemical contact between the larvae and the substrate [26]. The signaling molecules, i.e., Gigasin-6 isoform X1 and/or X2, CGDSP, and CGS12P, identified in this study showed amino acid residues, sequence insertions, and deletions that were unique in *C. gigas* in comparison with other organisms ). This may contribute to the specificity of the conspecific cue-recognition processes in *C. gigas* larval settlement. The ability of CGSPPC to induce settlement, either individually or collectively, highlights the important role of PTMs in oyster chemical signaling for conspecific recognition. The major settlement-inducing component in CGSPPC, which is Gigasin-6 isoform X1 and/or X2, is characterized as a phosphorylated sialoglycoprotein with varied *N*- and *O*-glycan specificities. It showed positive binding to WGA lectin which was previously reported to mediate settlement in *C. gigas* [23,24,26]. It may also function in settled juveniles as an attractant for other non-settled larvae to commence settlement. All these properties of Gigasin-6 isoform X1 and/or X2 provide support to previous findings [26], i.e., that multiple glycans may be necessary to produce high-avidity binding interactions during larval settlement induction [67] and that among these glycans, GlcNAc and Sialic residues may occupy the highest ratio of these sugar moieties as evidenced by an intense band pattern of Gigasin-6 isoform X1 and/or X2 that has bound to WGA lectin resin. The differential effects of sugar types on the attachment to a substrate until settlement suggests that such ratios within the settlement-inducing cue could determine the maximum adhesion of a settling larva on a substrate [68]. Moreover, CGDSP together with other isolated IDPs were also demonstrated in this study as co-factors that could contribute to strengthening the inducing signal via phosphorylation crosstalk with an unidentified CGSPPC-recognizing larval receptor. This suggests that the putative larval receptor may also be a phosphoprotein.

This present study highlights the importance played by PTMs in processes such as phosphorylation which could contribute to the rapid cellular response of oyster larvae in finding suitable substrates similar to those reported in cypris larvae [69]. This observation on the importance of phosphorylation is consistent with the report of Thiyagarajan et al. [69,70] on barnacles. Thiyagarajan et al. [70] hypothesized that protein phosphorylation is involved in regulating cypris larval metamorphosis. Exposure to a highly phosphorylated settlement cue activated phosphatase in searching cyprids which eventually led to a series of biochemical changes, attachment, and metamorphosis [69]. Furthermore, the rapid response to environmental cues and metamorphic morphogenesis of oyster larvae could be regulated by the presence of phosphorylated [69] and glycosylated [23,24,26,68] settlement cues on substrates and may explain why marine invertebrates, such as *C. gigas,* appear to require little or no de novo gene action during the metamorphic induction process [71]. A previous study also supports this theory that extensive de novo synthesis of proteins is not necessary for the completion of metamorphosis [59] and which was found to be similar in other reported studies in marine invertebrates [70,71]. Such a rapid transition from pediveliger to postlarvae is to the advantage of these organisms that are particularly vulnerable to predation during this event [72]. In contrast, as shown in Figure 6B, gregarious conspecific cues reported in other benthic organisms such as the barnacle SIPC presents a less complex lectin–glycan interaction during settlement induction as it is specifically modulated by LCA/Con A-binding sugars such as mannose [46,73].

**Figure 7:**
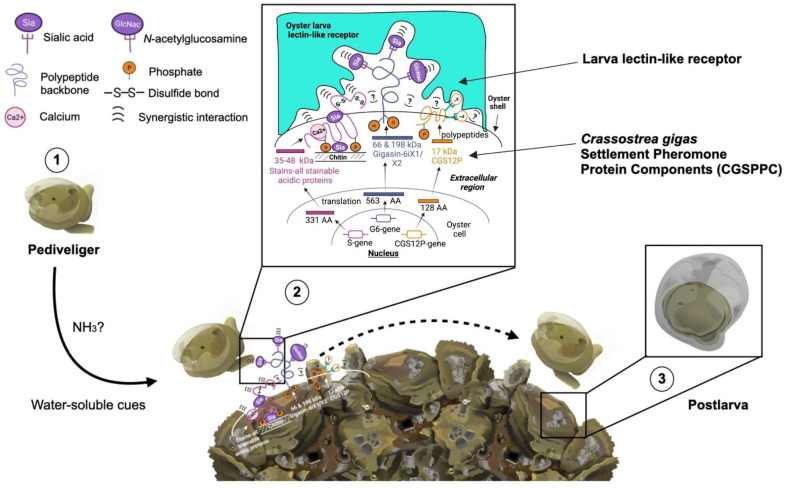
Schematic of the *Crassostrea gigas* Settlement Pheromone Protein Components (CGSPPC)-mediated larval settlement-induction mechanism. (**1**) As the oyster larva becomes a competent pediveliger, with a fully developed eye and foot, it begins to exhibit searching behaviors. This searching behavior is influenced by the detection of water-soluble cues from metabolites, including glycans, NH_3,_ and other amino acids, from bacteria or their conspecifics. (**2**) By locating the source of the inducing cue, the larva recognizes the amino acid groups, glycan moieties, and phosphate groups on the surface-bound cue, CGSPPC, that are found on shells of conspecific postlarvae and adult oysters. The noncovalent bond interaction between the lectin–glycan and/or phosphate groups in both the larva and substrate cue, i.e., shells of conspecifics, allows accumulation of transduced cues and creates a seemingly complex recognition system in *C. gigas*. This allows higher affinity, specificity, and complementarity between CGSPPC and its receptor to ensure greater selectivity during site selection. (**3**) Having accumulated enough signals from a suitable settlement site, a cascade of biochemical changes is triggered that leads to the attachment and its eventual metamorphosis into a postlarva. The postlarva develops newly formed gills and a new layer of an adult shell while its eye, foot, and velum disappear. This figure was partly created using BioRender.com (accessed on 25 July 2022).

Having discussed the individual and synergistic roles of the protein components within CGSPPC, the molecular basis of the conspecific cue-mediated larval settlement mechanism is proposed herein (Figure 7):

(1) When the oyster larva reaches a competent stage wherein the eye and the foot fully develop, the larva begins to exhibit searching behaviors such as swimming motions with a combination of foot extensions and localized crawling movements until it finds a suitable substratum, i.e., the shells of conspecifics [38]. The presence of dissolved water-soluble cues from the environment such as dissolved sugars [26,74], NH_3_ from metabolites, including glycans and amino acids from bacteria [75,76], or those from conspecifics [76] induces larval settlement behavior. It has been reported by Bonar et al., (1990) [77] that elevated levels of NH_3_ produced by a dense assemblage of oysters such as those of an oyster bed trigger the oyster larva to swim in search of the source of the corresponding cue. (2) As the larva locates the source of the inducing cue, it uses its external chemical receptors, such as those found in the velum and foot, to transduce the surface-bound cues it encounters on the substrata [17]. It then recognizes the amino acid groups, sugar moieties, and phosphate groups attached to the CGSPPC both from the newly attached juveniles and adult oysters. In this study, the settlement-inducing activity of CGSPPC was concentration-dependent implying that as the larva encounters more of its conspecifics, an increased settlement rate was observed. Furthermore, the interaction between the CGSPPC-recognizing larval receptor and this conspecific cue is reversible since their interaction is characterized by the formation of noncovalent bonds [24,26]. This allows the oyster larva to discriminate cues and gather confirmatory signals from the putative settlement substratum. (3) As the larva continues to exhibit a searching behavior along this area, a point is reached at which enough levels of accumulated signals (in the form of amino acid/sugar/phosphate groups) from the settlement-inducing cue, CGSPPC, trigger a cascade of biochemical changes that leads to the attachment and its eventual metamorphosis into a postlarva. In this rapid metamorphic change, the larva experiences the irreversible physiological process that includes a change from a swimming larva to a sessile juvenile, including loss of the velum, eyespot, and foot; development of gills, and production of an adult shell [9].

These present findings show that the induction of conspecific cue-mediated larval settlement in *C. gigas* requires an interplay of different amino acid groups, disulfide bonds, glycans, and phosphorylation crosstalk for recognition. On the other hand, the receptor that could recognize CGSPPC involves an unidentified WGA lectin-like larval receptor and a phosphoprotein. However, whether these sugar moieties and phosphate groups found in the components of CGSPPC could bind to the same larval receptor or not remains to be clarified. The noncovalent bond interaction between the lectin–glycan and/or phosphate groups in both larva and surface-bound cues from substrates, i.e., shells of conspecifics, allows accumulation of transduced cues and creates a seemingly complex recognition system in *C. gigas*. This facilitates higher affinity, specificity, and complementarity between CGSPPC and its receptor to ensure greater selectivity during site selection. Moreover, previous findings [26] and this present study showed that in the absence of a settlement cue (seawater only), oyster larvae do not settle and undergo metamorphosis [26]. The metamorphic process that larvae need to undergo requires a lot of energy and varied morphogenetic changes. Thus, this study supports the current hypothesis that oyster larvae actively respond to a wide range of variables or cues that may provide information to secure a site appropriate for its post-settlement growth and survival [23,78]. Oysters may seem simple, but this study provides insights into the complex molecular mechanisms governing oyster substrate selection that may indicate their evolutionary success as benthic organisms. Continued study on CGSPPC and its corresponding oyster larval receptor will advance our understanding of their evolution, population dynamics, and chemical communication. Lastly, these results may find application in the development of oyster aquaculture by using this extracted compound as a surface-bound attractant to collect wild and hatchery-grown oyster larvae which could help recover declining marine species; this compound could also find application as a target of anti-fouling agents on man-made structures.

## 4. Materials and Methods

### 4.1. Spawning and Larval Culture

The adult Pacific oysters (*Crassostrea gigas*) used in this experiment were purchased from Konagai Fisheries Cooperative, Nagasaki, Japan. The method for artificial fertilization and larval culture was based on Sedanza et al. [26,59]. Two to four male and five to eight female adult oysters were used as broodstocks for gamete stripping. The *C. gigas* larvae were cultured in 10-L filtered sea water-filled tanks under dark conditions with constant aeration. The seawater was renewed daily, and the larvae were fed with *Chaetoceros calcitrans* (10,000–50,000 cells/mL/day) from day 1 to day 5, a combination of *C. calcitrans* (25,000 cells/mL/day) and *Chaetoceros gracilis* (25,000 cells/mL/day) from day 6 to day 10, and then *C. gracilis* (50,000 cells/mL/day) from day 11 onward during the culture period. The water quality parameters during the larval culture period were salinity (30–32 psu), pH (8.02–8.08), and temperature (25–27 °C). The seawater was renewed daily prior to feeding. At least ten batches of different broodstocks were used for spawning to obtain larvae for the different settlement assays. Competent pediveligers in settlement assays were used when they reached an average shell height of 322 μm [26].

### 4.2. Extraction of Shell Matrices

Field observations on oyster reef structures indicate that oysters tend to attach to any part of the adult conspecific shells, whether it be on the outer surface or the underside. Hence, whole adult shell samples were crushed into smaller pieces and used for shell matrix extraction. The preparation of the *C. gigas* crude shell EDTA (CgSE) extract was carried out according to Sedanza et al. [26]. Shells from freshly shucked oysters (*C. gigas*) were thoroughly cleaned and washed with tap water to remove adhering epibionts and traces of muscle tissues, and then dried. The cleaned shells were then crushed with a hammer until shell chips of about 0.5 to 1.0 mm in size were collected. These shell chips (150 g) were decalcified with 1 L of 0.8 M Ethylenediaminetetraacetic acid (EDTA, 99% purity, Nacalai Tesque, Inc., Kyoto, Japan, pH 8.0) for 60 h at 4 °C with continuous agitation. The supernatant was collected by centrifugation at 10,000× *g* for 60 min at 4 °C. Then, it was subsequently filtered and dialyzed against distilled water at 4 °C for 3 days. The crude *C. gigas* shell EDTA-soluble extract (CgSE) was lyophilized, and the resultant powder was dissolved in as small a volume as possible of distilled water. This extract form is herein called Freeze-dried CgSE. Alternatively, another batch of dialyzed supernatant extract inside a dialysis tube was air-dried at a cool room temperature until the volume was reduced and the supernatant concentrated. This extract form is herein called Air-dried CgSE. The Freeze-dried (FD)-CgSE extract was used in all larval settlement assays while the Air-dried CgSE extract was used to compare with the FD-CgSE extract to check for protein stability during the extraction process. These two forms of CgSE extracts were stored at −20 °C until further use. The protein content of the extract was quantified by a BCA assay kit (Pierce, Thermo Fisher Scientific, Rockford, IL, USA) according to the manufacturer’s protocol. A gram of shell chips could yield 500 μg of CgSE extract.

### 4.3. First Protein Separation Approach

#### 4.3.1. Fractionation by Ultrafiltration

Ultrafiltration: Freeze-dried CgSE was fractionated into three molecular range fractions: >100K, >50K, and <50K molecular weight cut-off (MWCO) CgSE. This was carried out by separating CgSE into >100K and < 100K MWCO fractions using an Amicon ultra-15 ultrafiltration tube (100,000 MWCO, Merck Co., Ltd., Darmstadt, Germany). Then the <100K fraction was applied to a Vivaspin 20 ultrafiltration tube (50,000 MWCO PES, Sartorius, Gloucestershire, United Kingdom) to separate it into >50K and <50K MWCO fractions. All the fractionated samples were centrifuged at 2500× *g* for 40 min.

Larval Settlement Assay: Fractionated samples were subjected to a larval settlement assay at various extract amounts of 1 and 100 μg that were coated separately on the base of 6-well plates. Larval settlement assay was performed according to Sedanza et al. [26]. In brief, ten larvae were released into each pre-coated well plate filled with 10 mL of filtered seawater (FSW). The larval settlement was confirmed by the number of individuals that metamorphosed to postlarvae within 24 h. Post-larva was confirmed under a microscope as individuals that secreted cement substances or those with post-larval shell growth. All succeeding larval settlement assays were conducted in a dark environment at 27 ± 1 °C in an incubator. Another batch of larvae was also released into the well plates containing filtered seawater (FSW) as the control. The larval settlement assays were repeated twice using different batches of larvae with three replicates at each trial (*n* = 6). All data were presented as the mean ± standard deviation (SD).

#### 4.3.2. Gel Filtration of >50K CgSE

Gel filtration chromatography: The >50K CgSE active fraction (6 mg) was applied to a Superdex 200 10/300 GL column (GE Healthcare, Tokyo, Japan) equilibrated with 0.15 M NaCl using a 500 μL loop and eluted with the same buffer at a rate of 0.25 mL min^−1^, using an AKTA explore 10S- FPLC system (GE Healthcare, Tokyo, Japan). A total of 35 tubes of 0.5 mL were collected and pooled into six molecular range fractions: F1, F2, F3, F4, F5, and F6.

Larval Settlement Assay: The Gel filtration-eluted fractions were subjected to a larval settlement assay at various extract amounts of 1 μg (*n* = 3), 10 μg (*n* = 6), and 50 μg (*n* = 5). Larval assay replications for each extract amount varied due to the limited protein yield from the eluted fractions. Several 6-well plates were pre-coated with the test extracts and the larval assay was carried out as described earlier. All data were presented as the mean ± standard deviation (SD).

SDS-PAGE Analysis: For SDS-PAGE analysis on the active fractions >50K CgSE, F2, and F3, 10 µg of each sample under reducing conditions were loaded in each lane on a 10% acrylamide gel for 1 h at 200 volts [79]. A pre-stained Protein Standard marker (Broad range, Bio-Rad, Hercules, CA, USA) was also loaded on the gel. Following SDS-PAGE, all the protein bands were visualized by the Stains-all staining method following the manufacturer’s instruction manual.

#### 4.3.3. Separation of F2 Proteins

Protein separation by SDS-PAGE: sixty μg of the active fraction, F2, from gel filtration was applied per lane and resolved in duplicate on 10% SDS-PAGE gels under non-reducing and reducing (Laemmli) conditions at 200 V for 1 h [79]. One gel was stained with Stains-all (Sigma-Aldrich, Buchs, Switzerland), following the manufacturer’s protocol, to reveal the positions of acidic and phosphorylated proteins. The unstained duplicate gel was equilibrated in a transfer buffer (containing 10% methanol, 5 mM Tris, 38 mM Glycine, pH 8.3) and shaken for 15 min. A PVDF membrane (Sequi-Blot, Bio-Rad, Hercules, CA, USA), with the same size as the duplicate gel, was immersed in 100% methanol until it became translucent. Subsequently, the membrane was equilibrated in a transfer buffer following the same procedure as earlier described. Next, the equilibrated duplicate gel was transferred electrophoretically onto the PVDF membrane for 150 min at 0.8 mA/cm^3^. Following the transfer, the membrane was immersed in a container containing ultrapure water and shaken for 15 min. This step was repeated six times. Then, the six major bands of F2 under non-reducing conditions (35, 38, 43, 48, 60 kDa) and three major bands under reducing conditions (45, 48, 53 kDa) were cut out using the stained marker (Pre-stained SDS-PAGE Standards Broad range, Bio-Rad, Hercules, CA, USA). The Stains-all-stained gel was used as a guide. This process was repeated several times until the number of PVDF strips containing the isolated polypeptides was enough for larval settlement assay. Isolated polypeptide bands that showed settlement-inducing activity were further subjected to Edman sequencing for identification.

Larval settlement assay: three PVDF strips containing an isolated polypeptide were fastened side-by-side to the base of each well on a 6-well plate. This step was repeated for all polypeptides. Each well was then filled with 10 mL FSW followed by the immersion of ten larvae. The succeeding steps for the larval assay were carried out as described earlier. These settlement assays were repeated twice using different batches of larvae with three replicates at each trial (*n* = 6). All data were presented as the mean ± standard deviation (SD).

### 4.4. Protease and TFMS Treatment

To characterize further the Stains-all-stainable acidic protein components through biochemical analysis, CgSE was treated with various proteases and Trifluoromethanesulfonic acid (TFMS) for chemical deglycosylation. Two hundred micrograms of CgSE was treated separately by three proteases and performed according to the manufacturer’s protocol. Twenty microliters of Trypsin (Trypsin singles, Proteomics grade, Sigma-Aldrich, Missouri, USA) was added to the CgSE dissolved in 10% ACN, 25 mM NH_4_HCO_3_, to constitute a 1:100 enzyme-to-protein ratio. Twenty microliters of Chymotrypsin (Roche, Mannheim, Germany) was added to the CgSE dissolved in 100 mM Tris-HCl, and 10 mM CaCl_2_, pH 7.8. The ratio of Chymotrypsin enzyme to protein was at 1:100. Twenty-five microliters of Asp-N (Roche, Mannheim, Germany) was added to the CgSE dissolved in 50 mM sodium phosphate buffer, pH 8.0. The ratio of Asp-N enzyme to protein was at 1:200. All the protease digests were incubated at 37 °C for 16 h. For the TFMS treatment assay (Wako Pure Chemicals Co., Osaka, Japan), equal amounts of CgSE (0.5 mL) corresponding to 40 µg of protein and TFMS (0.5 mL) were mixed and incubated for 1 h on ice, following the manufacturer’s instruction manual. The TFMS digest was dialyzed against distilled water to remove excess TFMS in the mixture as recommended by Vasquez et al. [23]. Forty micrograms from each protease and TFMS digest were loaded on a 12% polyacrylamide gel and analyzed for SDS-PAGE under reducing conditions [79] following the steps described earlier. All the protein bands were visualized under Stains-all staining. These protein bands were further subjected to proteomic analysis.

Endoproteinase, Glu-C (*S. aureus* V8 protease, Mass spectrometry grade, 20 µg/mL, Pierce, IL, USA) was added to 10 µg of >50K CgSE dissolved in 25 mM NH_4_HCO_3_ under different enzyme-to-protein ratios of 1:100, 1:50, and 1:10. The Glu-C digests were incubated at 37°C for 16 h, loaded on a 12% polyacrylamide gel, and analyzed for SDS-PAGE under reducing conditions [79] following the steps described earlier. All the protein bands were visualized under Stains-all staining.

For the SDS-PAGE analysis of >50K CgSE and its active gel filtration-eluted fractions, i.e., F2 and F3, 10 µg of each sample under reducing conditions were loaded in each lane on a 10% acrylamide gel for 1 h at 200 volts [79]. A pre-stained Protein Standard marker (Broad range, Bio-Rad, Hercules, CA, USA) was also loaded on the gel. Following SDS-PAGE, all the protein bands were visualized under Stains-all staining.

### 4.5. Second Protein Separation Approach

#### 4.5.1. Wheat Germ Agglutinin (WGA) Agarose-Bound Lectin Affinity Chromatography

The previous protein purification steps in this study showed that the settlement-inducing effect of the target proteins decreased as they were further separated into each polypeptide. Hence, a change in strategy was performed by subjecting crude CgSE to another fractionation by ultrafiltration, this time using a 50K MWCO centrifugation tube to separate it into <50K and >50K fractions. The >50K CgSE fraction was applied to lectin affinity chromatography on WGA-agarose-bound resin. WGA-agarose-bound lectin with a protein concentration of 7 mg/mL gel and binding capacity of >8 mg GlcNAc/mL gel was obtained from Vector Laboratories (Burlingame, CA, USA). A 1.0 M NaCl buffer system was used for WGA lectin column binding. A modified method of Yang and Hancock was adopted in the preparation of lectin affinity columns [80]. The WGA affinity column was prepared by adding 0.5 mL of the corresponding agarose-bound lectin to an empty 0.8 mL Micro Bio-Spin chromatography column (Bio-Rad, Hercules, CA, USA). The WGA agarose-bound spin column was equilibrated with 10 column volumes of binding buffer containing 0.05 M Tris-HCl, 1.0 M NaCl, pH 8.0. Subsequently, 3 mg of >50K CgSE was loaded on the column. After 10 min. of sample application, the unbound proteins were eluted with 6 column volumes of the binding buffer and collected and pooled as the Unbound Fraction (UF). Then, the captured glycoproteins, which bound to the immobilized WGA lectin, were eluted using 10 column volumes of a buffer containing 0.5 M GlcNAc, 0.05 M Tris-HCl, 1.0 M NaCl, pH 8.0. The eluted fraction was collected as the pooled, Bound Fraction (BF) component. Washing of the column was done by a repetition of loading binding buffer in the spin column and draining it out by centrifugation at 5000 rpm for 30 s for 10 times. The process of sample application and elution was repeated twice to gather enough samples for larval settlement assays and biochemical analyses. The UF and BF components were dialyzed overnight against 2–3 times buffer change containing 20 mM Tris-HCl (pH 8.0). The dialyzed UF and BF inside the dialysis tubes were subsequently concentrated by air-drying at cool room temperature until the original volume was reduced to a small volume. These were then stored at −20 °C until further use.

CgSE, >50K CgSE, UF, and BF protein samples were subjected to a larval settlement assay at various extract amounts of 0, 0.5, 5, and 50 μg that were coated separately on the base of 6-well plates. Each treatment was completed in triplicates. The succeeding steps for the larval assay were carried out as described earlier.

#### 4.5.2. SDS-PAGE Analysis of WGA Affinity Chromatography-Eluted Fractions

The CgSE, >50K CgSE, UF, and BF protein samples were analyzed on a 12% TGX FastCast polyacrylamide gel (1.0 mm × 10 well) (Bio-Rad, Hercules, CA, USA) with a loading amount of 10 μg for each sample. Pre-stained molecular weight markers (Precision Plus Protein Dual Xtra, Bio-Rad, Hercules, CA, USA) were loaded alongside the samples. Samples were suspended in 2× Laemmli sample buffer (125 mM Tris-HCl (pH 8.5), 20% glycerol, 4% SDS, 10% 2-mercaptoethanol, and 0.0025% bromophenol blue) under reducing conditions in the presence of 2-mercaptoethanol [79]. SDS-PAGE was run using a Mini-Protean Tetra System (Bio-Rad, Hercules, CA, USA) at 200 volts for 40 min. The gels were stained with QC-Colloidal Coomassie stain (Bio-Rad, Hercules, CA, USA), Silver stain (Silver Stain MS Kit, Fujifilm Wako Pure Chemical Corporation, Osaka, Japan), and Stains-all for putative calcium-binding and highly acidic, phosphorylated proteins [52,56]. Gel staining was performed according to the manufacturer’s protocol. The gel images for figure presentation and band molecular weight determination were captured using a GELSCAN^®^ laser scanner (iMeasure, Nagano, Japan). For the purposes of figure presentation, another set of colored gel images was captured using a digital camera. The estimated molecular weight of each protein band in a sample was calculated using Gel-Pro Analyzer 4.0 software.

### 4.6. Glycoprotein Detection

Ten micrograms of the eluted WGA affinity chromatography components, i.e., the UF and BF samples, were deglycosylated using 1 μL PNGase F (0.5 mU/μL, Takara Bio Inc., Kyoto, Japan) under denaturing conditions. Another batch of samples was treated with a mixture of 2 μL O-glycosidase (40,000 U/μL), 2 μL PNGase F (0.5 mU/μL), and 2 μL Neuraminidase (50 U/μL) (New England BioLabs Inc., Massachusetts, USA). All enzyme reactions were conducted with an overnight incubation at 37 °C for 20 H following the manufacturer’s protocol. Subsequently, the samples (1) >50K CgSE, (2) untreated, and (3-4) deglycosylated WGA affinity chromatography-eluted fractions (UF and BF), were subjected to Cys-β-propionamidation following the method of Unno et al. (2018) [81] for the mass spectrometry analysis preparation. In brief, 5 μL of each protein (10 μg) sample was added to 5 μL of 2× SDS-sample buffer for Cys alkylation (125 mM Tris-HCl (pH 8.5), 20% glycerol, 4% SDS, 10% 2-mercaptoethanol, and 0.0025% bromophenol blue), and the protein solution was incubated at 95 °C for 10 min. Five microliters of 7 M acrylamide was added to the reduced protein solution and held for 1 h at room temperature. The resulting propionamidated protein sample solutions (15 µL/well) were applied to a 12% TGX FastCast polyacrylamide gel and SDS-PAGE [79] was performed following the same conditions as described earlier. The gel was stained with Pro-Q Emerald 488 glycoprotein gel stain kit (Invitrogen, Carlsbad, USA) following the manufacturer’s protocol. The stained gel was viewed and photographed using a CCD camera from FluoroPhorestar 3000 (Anatech, Tokyo, Japan). For protein identification through mass spectrometry analysis, the gel was double stained with QC-Colloidal Coomassie stain following the manufacturer’s protocol.

For the glycoprotein detection, enzymatic deglycosylation with PNGase F only and with a mixture of *O*-glycosidase, PNGase F, and Neuraminidase was performed and resolved on a 12% SDS-PAGE gel, after which the gel was stained with Pro-Q Emerald for detection of glycoproteins and double-stained with QC-CC stain for further mass spectrometry analysis.

### 4.7. Phosphoprotein Detection

CgSE in its freeze-dried (non-reducing and reducing conditions) and air-dried (reducing condition) forms, >50K CgSE, UF, and BF component samples were analyzed for phosphoprotein detection. Ten micrograms of each sample were applied to SDS-PAGE under the same conditions as described earlier (Methods 4.6), except for the omission of the Cys-β-propionamidation step and with some slight modifications. In brief, after SDS-PAGE, the gel was stained by Pro-Q Diamond Phosphoprotein gel stain (Invitrogen, Carlsbad, CA, USA) according to the product’s manual. The gel was viewed and photographed using a CCD camera from FluoroPhorestar 3000 (Anatech, Tokyo, Japan).

### 4.8. Mass Spectrometry Analyses

The CgSE extract and its varied fractionated and purified samples that were selected for mass spectrometry analyses were subjected to Cys-β-propionamidation before SDS-PAGE following the procedures described earlier (Methods 4.6). After SDS-PAGE, the method reported by Yamaguchi [82] was followed for the in-gel enzymatic digestion, peptide extraction from the gel, and sample loading onto a matrix-assisted laser desorption/ionization (MALDI) target plate. MS and MS/MS spectra were obtained using a matrix-assisted laser desorption/ionization (MALDI) quadruple-ion trap (QIT) time-of-flight (TOF) mass spectrometer (AXIMA Resonance, Shimadzu, Kyoto, Japan) with 2, 5-dihydroxybenzoic acid (DHBA, Shimadzu, Kyoto, Japan) as the matrix in positive mode. MALDI-QIT-TOF mass spectra were externally calibrated using human angiotensin II (m/z 1046.54) and human ACTH fragment 18–39 (m/z 2465.20) in a ProteoMass Peptide and Protein MALDI-MS Calibration Kit (Sigma-Aldrich, St. Louis, MI, USA). Modification of the N-terminal peptide was analyzed by PMF and an MS/MS ion search using MASCOT ver. 2.3 (Matrix Science, London, UK) with an original database from (*C. gigas* 65,722 protein sequences; https://www.ncbi.nlm.nih.gov/Taxonomy/Browser/wwwtax.cgi?id=29159 accessed on 8 April 2022 in our MASCOT server). The search parameters used for PMF were the following: enzyme, Trypsin; fixed modifications, propionamide (C); variable modifications, oxidation (HW and M); mass values, monoisotopic; peptide mass tolerance, +/−0.5 Da; peptide charge state, 1+; and a maximum number of missed cleavage sites, 1. The search parameters used for the MS/MS ions search were as follows: enzyme, Trypsin; fixed modifications, propionamide (C); variable modifications, oxidation (HW and M); mass values, monoisotopic; peptide mass tolerance, +/−0.3 Da; fragment tolerance, +/−0.2 Da; and max missed cleavages, 1. A protein score (PMF) > 51 and an individual ion score (MS/MS ions search) >14 were considered significant (*p* < 0.05).

### 4.9. Localization of N-glycosylation Sites by Mass Spectrometry Analysis

Mass spectrometric localization of the *N*-glycan sites of Gigasin-6 isoform X1 and/or X2 (66 kDa) was conducted by comparing the MS and MS/MS spectra profiles of the deglycosylated (DG2/64 kDa) and glycosylated (B2/66 kDa) bands of interest, i.e., with and without PNGase F treatment, respectively. The method for the determination of actual *N*-glycosylation sites was adopted from León et al. [58] with slight modifications. In brief, before SDS-PAGE, the UF and BF components were treated with PNGase F, reduced, alkylated, and further processed as described earlier (Methods 4.6). Then, the gels were stained with QC-Colloidal Coomassie stain and subjected to in-gel enzymatic digestion and mass spectrometry analysis. The full-range MS spectra of both the glycosylated and deglycosylated bands were compared and searched for a newly appeared signal on the m/z region of the deglycosylated digest. This newly appeared signal corresponded to a positive mass shift of approximately 0.98 da for every previously glycosylated asparagine that was deaminated to aspartic acid. All newly appeared signals were further subjected to MS/MS and Collision-Induced Dissociation (CID, 250), and the resulting analyzed peptide fragments were analyzed against an error-tolerant search to confirm their identity with matched protein sequences reported in Gigasin-6 isoform X1 and/or X2 and those found in our MASCOT server. The search parameters were as follows: enzyme, Trypsin; fixed modifications, propionamide (C); variable modifications, oxidation (HW and M); mass values, monoisotopic; peptide mass tolerance, +/−0.5 Da; peptide charge state, 1+; and a maximum number of missed cleavage sites, 1. Alternatively, newly appeared signals from the deglycosylated digest that did not receive an error-tolerant search hit were manually annotated by matching observed MS/MS ion fragments with the Protein Prospector MS-Product program (https://prospector.ucsf.edu/prospector/cgi-bin/msform.cgi?form=msproduct (accessed on 31 March 2022)). Manual validation of the identified unique peptides took into account the assignment of the major peaks, the occurrence of uninterrupted y- or b-ion series of at least four 4 consecutive amino acids, the possible presence of a2/b2 ion pairs and immonium ions, and mass accuracy [83].

### 4.10. Characterization of Protein Sequences

Putative sequences identified through a mass spectrometry analysis were characterized using different bioinformatic tools. Signal peptide prediction was conducted with SignalP 6.0 [84]. Functional domains were searched for using the InterProscan and ScanProsite platforms [85,86]. SMART (simple modular architecture research tool) was used to allow the rapid identification and annotation of signaling domain sequences [87,88]. Transmembrane helices were predicted and proteins containing transmembrane helices lower than 18 or found within the signal peptide region were filtered out according to the Transmembrane Helices Hidden Markov Model (TMHMM) [89]. Phosphorylation sites in the sequences were predicted using NetPhos 3.1 [90]. *N*- and *O*-glycan sites were predicted using Net*N*Glyc-1.0 and Net*O*Glyc-4.0, respectively [91,92]. The prediction of intrinsically unstructured proteins and detection of low complexity regions (LCRs) were carried out using IUPred3 software [93]. Both the identified and unidentified groups of protein sequences in the mass spectrometry analysis were also Blastp-searched against nr in NCBI using default settings (https://blast.ncbi.nlm.nih.gov/Blast.cgi/ (accessed on 31 March 2022)). After mass spectrometry analysis, a homology search was performed on the unidentified Stains-all-stainable acidic proteins with a putative homolog in *C. gigas* to Folian-cv1 of *C. virginica* [40]. Multiple sequence alignments were conducted on various putative homolog groups using Clustal Omega (https://www.ebi.ac.uk/Tools/msa/clustalo/ (accessed on 31 March 2022)) [94].

### 4.11. Molecular Characterization of Gigasin-6 Isoform X1

To examine the mRNA expression pattern of the Gigasin-6 isoform X1 gene in the larvae before (pediveliger) and after conspecific cue induction (postlarvae), a larval settlement assay was conducted using the unfractionated CgSE sample and a quantitative real-time PCR (qRT-PCR) assay was performed.

Larval sample preparation was conducted following the method reported by Sedanza et al. [59] by using two batches of larvae: (1) Control, pediveliger larvae before conspecific cue settlement induction (which means no CgSE exposure), and (2) Postlarvae, conspecific cue-induced larvae (which means with CgSE exposure). Larvae were at 24 days post-fertilization (or 24 days of culture, DOC) when approximately 70–80% had developed an eyespot and started to crawl. To ensure that all the larvae were at a competent stage, larvae from multiple 10-L FSW-filled tanks were sieved over a 300 μm mesh-covered tip nestled on a glass bowl filled with FSW. They were carefully washed in Millipore FSW (0.22 μm) and transferred to several 3-L beaker containers newly filled with FSW. After filtration, more than 95% of the larvae were verified under the microscope to have reached the pediveliger stage. The larval samples were then divided into two batches. Beakers containing the control batch of competent pediveliger larvae were siphoned over a 300 μm mesh-covered tip nestled on a glass bowl filled with FSW, carefully washed with distilled water, and collected as a pooled Pedi sample. Approximately 100,000 individuals of pooled pediveliger larvae (183 mg wet weight) in a microcentrifuge tube were treated immediately with RNAlater Stabilization Solution (Invitrogen, Vilnius, Lithuania) for 24 h at 4 °C following the manufacturer’s protocol. After this, the RNAlater was removed, and the pooled Pedi batch was stored at −80 °C prior to the subsequent experiments. For the PL batch, the method previously described by Sedanza et al. [26] was adopted for the settlement induction of oyster larvae with CgSE. Briefly, 6-well plates were pre-coated with 50 μg CgSE and subsequently filled with 10 mL of filtered seawater per well. Twenty larvae were released in each well and induced to settle on the same day as the collection of the Pedi larvae, i.e., at 24 DOC.

Postlarvae were confirmed under the microscope as individuals that secreted cement substances or those with postlarval shell growth. Approximately 80% settlement success was observed after induction. The remaining larvae that were not induced to settle were found actively swimming after 24 h. No mortality was observed. All postlarvae that metamorphosed after 24 h were carefully removed from the 6-well plate bases. After removal of the postlarvae from each well, they were pipetted out to a 300 μm mesh-covered tip nestled on a glass bowl filled with FSW, washed gently several times with distilled water to remove any traces of the protein extract, and were transferred to a clean microcentrifuge tube. After the removal of the excess liquid on the tube, the postlarvae were immediately treated with RNAlater. This whole process was repeated several times until all the samples were collected. After 24 h, a similar procedure to that of the Pedi batch was carried out, wherein all the postlarvae were pooled and RNAlater was removed. Approximately 3000 individuals of postlarvae, equivalent to about 100 mg of pooled sample, were stored at −80 °C until further use.

Quantitative Real-Time PCR (qRT-PCR) analysis on Gigasin-6 isoform X1 (G6iX1) was determined and compared in both oyster larval stages. PCR primers for Gigasin-6 isoform X1 were designed using the NCBI primer blast tool (https://www.ncbi.nlm.nih.gov/tools/primer-blast/ (accessed on 25 October 2021)) and the oligoanalyzer tool of Integrated DNA Technologies (IDT) (https://sg.idtdna.com/calc/analyzer (accessed on 25 October 2021)). A forward primer (5′–AGGCTCTCGGGGTCAAGGACA–3′) corresponding to the 233-253 bp region of G6iX1 and a reverse primer (5′-GAGTAGAATCTGGGTTGTTTG–3′) corresponding to the 362-381 bp region were purchased from Life Technologies Japan Ltd. The expected length of the target G6iX1 product was 150 bp. The total RNA from both larval stage samples was reverse-transcribed following the method of Gao et al. [95]. In brief, all RNA samples were measured for quantity and quality by a NanoDrop ND-1000 spectrometer (Thermo Fisher Scientific, Wilmington, DE, USA). cDNA was synthesized by treating the total RNA sample (500 ng) with oligo-dT primer CDS-BR (5-GGCCACGCGTCGACTAGTAC(T)16–3′), random primer (5-NNNNNNNNN-3′), and M-MLV reverse transcriptase (Promega, Madison, WI, USA). qPCR assay was conducted using Power SYBR Green PCR Master Mix and a QuantStudio 3 Real-Time PCR System (Applied Biosystems, Thermo Fisher Scientific, Rockford, IL, USA) following the manufacturer’s instructions. Thermocycling was performed as follows: enzyme activation for 10 min at 95 °C, denature step for 15 s at 95 °C, and annealing and extension for 1 min at 60 °C for 40 cycles. Expressions for G6iX1 were normalized to that of the reference gene *elf* (elongation factor 1) for each larval stage sample collection. The reference gene primer used was as reported by Huan et al. [96]. The mRNA expression levels were quantified by the standard curve method, and the relative mRNA expression levels were calculated by the ratio of G6iX1 to the *elf* reference gene. All data were presented as the mean ± standard deviation (SD). This experiment was conducted in triplicate.

### 4.12. Statistical Analyses and Visualization

The settlement percentage (%) was calculated based on the number of settled larvae over the total number of larvae in each well multiplied by 100. These percentages were presented as arithmetic means with standard deviation (SD). Data were analyzed using quasi-binomial generalized linear models (GLM). All larval settlement statistical tests were performed in RStudio (R-project.org, version 4.1.3) [97].

The comparison of the relative Gigasin-6 isoform X1 mRNA expression levels between pediveliger and postlarvae samples were analyzed using the Student *t*-test and was determined using the GraphPad 9 software program (GraphPad Prism, Software Inc., San Diego, CA, USA). All figures in this study were created using BioRender.com (accessed on 25 July 2022) and the GraphPad 9 software program.

## 5. Conclusions

This study reports a novel role of a macromolecular assembly of shell matrix proteins that acts as *Crassostrea gigas* Settlement Pheromone Protein Components (CGSPPC), which is demonstrated as the biological cue responsible for gregarious settlement on conspecifics. These isolated matrix proteins from the adult oyster shells include Gigasin-6 isoform X1 and/or X2, *Crassostrea gigas* Surface protein P12p-like (CGS12P), and several Stains-all-stainable acidic proteins, the most dominant of which was a 48 kDa band, putatively identified as *Crassostrea gigas* Dentin sialophosphoprotein (CGDSP). Each protein factor may individually induce larval settlement, but evidence also shows that they could synergistically interact with each other to form a macromolecular assemblage within the shell to create a stable and strong settlement-inducing signal. Some key factors that are attributed to their ability to induce settlement, either individually or collectively, are the presence of unique amino acid groups and post-translational modifications that may play important roles in oyster chemical signaling for conspecific recognition. Gigasin-6 isoform X1 and/or X2, which showed positive binding to WGA lectin, is considered the major settlement-inducing cue component. Gigasin-6 isoform X1 and/or X2 plays a role in larval settlement induction on adult shells and may also function in postlarva–larva settlement interactions. CGDSP together with other Stains-all-stainable acidic proteins are also co-factors that could contribute to strengthening the settlement-inducing signal via phosphorylation crosstalk with a CGSPPC-recognizing larval receptor. CGDSP and some Stains-all-stainable acidic proteins isolated in this study require disulfide bonds to stabilize its settlement-inducing effects and may play a role in providing the structural framework for other shell matrix proteins to anchor within the settlement protein components and provide protection to its multiple binding partners. The role of CGS12P as a co-factor in settlement induction warrants further investigation. Furthermore, conspecific cue-mediated larval settlement induction in *C. gigas* presents a complex system that requires an interplay of different amino acid groups, disulfide bonds, glycans, and phosphorylation crosstalk for conspecific recognition. On the other hand, the receptor that could recognize CGSPPC may involve a WGA lectin-like larval receptor and a phosphoprotein.

## Figures and Tables

**Table 1:** Identification of the EDTA-soluble shell matrix proteins of *Crassostrea gigas* by Peptide Mass Fingerprinting Analysis in the unbound and bound fractions eluted after WGA affinity chromatography.

Band No.	Protein Name ^a^	Accession Number ^a^	Theoretical Mass (kDa)	Observed Mass (kDa)	PS/NMP ^b^	SC ^c^ (%)
**Unbound fraction components**						
UB1	Gigasin-6 isoform X2	XP_011449648.2	63	198	79/15	20
	Gigasin-6 isoform X1	XP_011449647.2	64	198	78/15	19
UB2	Gigasin-6 isoform X2	XP_011449648.2	63	66	85/15	21
	Gigasin-6 isoform X1	XP_011449647.2	64	66	84/15	20
UB3	Surface protein P12p-like	XP_034321529.1	15	17	80/8	37
	Surface protein P12p-like	XP_034319527.1	15	17	80/8	37
S1	Unidentified Stains-all-stainable acidic protein	N/A	N/A	48	N/A	N/A
S2	Unidentified Stains-all-stainable acidic protein	N/A	N/A	43	N/A	N/A
**Bound fraction components**						
B1	Gigasin-6 isoform X1	XP_011449647.2	64	198	97/17	21
	Gigasin-6 isoform X2	XP_011449648.2	63	198	96/17	22
B2	Gigasin-6 isoform X1	XP_011449647.2	64	66	63/13	18
	Gigasin-6 isoform X2	XP_011449648.2	63	66	63/13	18

^a^ Protein name and accession number according to NCBI database, ^b^ Protein score (PS) and the number of matched peptides (NMP) obtained from Mascot search, ^c^ Percentage of sequence coverage of identified peptides related to the corresponding sequence in the database.

**Table 2:** MS/MS-based identification and characterization of the protein band containing the determined *N*-glycosylation sites of Gigasin-6 isoform X1 and/or X2 in the WGA column-bound fraction (BF) after treatment with PNGase F.

Band No.	Protein Name ^a^	Accession Number ^a^	Theoretical Mass (kDa)	Observed Mass (kDa)	PS/NMP ^b^	SC ^c^ (%)
**Bound fraction components**						
**Deglycosylated**						
DG1	Gigasin-6 isoform X1	XP_011449647.2	64	196	73/14	21
	Gigasin-6 isoform X2	XP_011449648.2	63	196	72/14	22
DG2	Gigasin-6 isoform X1	XP_011449647.2	64	64	91/16	21
	Gigasin-6 isoform X2	XP_011449648.2	63	64	90/16	22

^a^ Protein name and accession number according to NCBI database, ^b^ Protein score (PS) and the number of matched peptides (NMP) obtained from Mascot search, ^c^ Percentage of sequence coverage of identified peptides related to the corresponding sequence in the database.

## Data Availability

The authors declare that all other data supporting the findings of this study are available within the paper and its supplementary information files.

## Supplementary Materials

The following supporting information can be downloaded at: https://www.mdpi.com/article/10.3390/ijms23179816/s1. References [11,40,44,46,55,56,57,58,60,79,83,98,99,100] are cited in the supplementary materials.
