# Supplementary material for: Identification and Characterization of the Larval Settlement Pheromone Protein Components in Adult Shells of Crassostrea gigas: A Novel Function of Shell Matrix Proteins
Source: Int J Mol Sci. 2022 Aug 29;23(17):9816. doi: 10.3390/ijms23179816 (PMC9456362; doi:10.3390/ijms23179816)
Supplement: Supplementary file 1 [file ijms-23-09816-s001.zip › ijms-1859319-supplementary.pdf]

SUPPLEMENTARY INFORMATION

Supplementary Information for

Identification and Characterization of the Larval Settlement Pheromone Protein Components in Adult Shells of *Crassostrea gigas*: A Novel Function of Shell Matrix Proteins

Mary Grace Sedanza[1,2,*], Asami Yoshida[1], Hee-Jin Kim[1], Kenichi Yamaguchi[1], Kiyoshi Osatomi[1], Cyril Glenn Satuito[1,3]

[1] Graduate School of Fisheries and Environmental Sciences, Nagasaki University, Nagasaki 852-8521, Japan.

[2] Institute of Aquaculture, College of Fisheries and Ocean Sciences, University of the Philippines Visayas, Miag-ao Iloilo 5023, Philippines.

[3] Organization for Marine Science and Technology, Nagasaki University, Nagasaki 852-8521, Japan

* Correspondence: M.G.S. (bb53419802@ms.nagasaki-u.ac.jp; mcsedanza@up.edu.ph); Tel.: (+81-95-819-2853)

This file includes:

Supplementary text: Results

Supplementary figures S1-S11

Supplementary tables S1-S2

Supplementary references S1-S14

TABLE OF CONTENTS

SUPPLEMENTARY TEXT 3
A. FIRST PROTEIN SEPARATION APPROACH 3
A.1 FRACTIONATION BY ULTRAFILTRATION 3
A.2 GEL FILTRATION OF >50K CGSE 3
A.3 SEPARATION OF F2 PROTEINS 3
B. MOLECULAR CHARACTERIZATION OF GIGASIN-6 ISOFORM X1 4
B.1 BIOINFORMATIC CHARACTERIZATION OF GIGASIN-6 ISOFORM X1 PROTEIN SEQUENCE 4
B.2 LOCALIZATION OF *N*-GLYCOSYLATION SITES BY MASS SPECTROMETRY ANALYSIS 4
C. MOLECULAR CHARACTERIZATION OF STAINS-ALL STAINABLE ACIDIC PROTEINS 5
D. MOLECULAR CHARACTERIZATION OF SURFACE PROTEIN P12P-LIKE 6
SUPPLEMENTARY FIGURES 7
FIG. S1 | SETTLEMENT PERCENTAGES OF *C. GIGAS* LARVAE ON DIFFERENT ULTRAFILTRATION FRACTIONS WITH VARYING AMOUNTS OF CGSE AFTER 24 H. 7
FIG. S2 | CHROMATOGRAM PROFILE OF FRACTIONS ELUTED FROM >50K CGSE GEL FILTRATION CHROMATOGRAPHY. 8
FIG. S3 | SDS-PAGE ANALYSIS AND LARVAL SETTLEMENT BIOASSAY AFTER >50K CGSE GEL FILTRATION CHROMATOGRAPHY. 9
FIG. S4 | SDS-PAGE PROFILE AND LARVAL SETTLEMENT BIOASSAY OF ACTIVE FRACTION F2 AND ITS ISOLATED POLYPEPTIDES. 10
FIG. S5 | CHROMATOGRAM PROFILE OF FRACTIONS ELUTED AFTER WGA AFFINITY CHROMATOGRAPHY UNDER 1.0 M NACL BUFFER SYSTEM. 11
FIG. S6 | MULTIPLE SEQUENCE ALIGNMENT OF GIGASIN-6 AND ITS ISOFORMS. 12
FIG. S7 | MULTIPLE SEQUENCE ALIGNMENT OF GIGASIN-6 ISOFORM X1 WITH HOMOLOGOUS SEQUENCES FROM OTHER BIVALVES. 13
FIG. S8 | MASS SPECTROMETRY ANALYSIS ON THE CRASSOSTREA GIGAS GIGASIN-6 ISOFORM X1 AND/OR X2 AND ITS TRIMER. 14
FIG. S9 | MULTIPLE SEQUENCE ALIGNMENT OF NCBI BLAST SEARCHED FOLIAN CV1 HOMOLOGS. 15
FIG. S10 | MOLECULAR CHARACTERIZATION OF *CRASSOSTREA GIGAS* SURFACE PROTEIN P12P-LIKE. 16
FIG. S11 | MULTIPLE SEQUENCE ALIGNMENT OF *CRASSOSTREA GIGAS* SURFACE PROTEIN P12P-LIKE WITH ITS HOMOLOGS 17
SUPPLEMENTARY TABLES 18
TABLE S1 | RESULTS OF PEPTIDE MASS FINGERPRINTING (PMF) ION SEARCH FOR PROTEIN IDENTIFICATION OF MATRIX PROTEINS IN THE *CRASSOSTREA GIGAS* SOLUBLE EDTA SHELL EXTRACT. 18
TABLE S2 | MS/MS-BASED IDENTIFICATION AND CHARACTERIZATION OF THE PROTEIN BAND CONTAINING THE DETERMINED *N*-GLYCOSYLATION SITES IN GIGASIN-6 ISOFORM X1 AND/OR X2. 24
SUPPLEMENTARY REFERENCES 25

Supplementary text: Results

A. First Protein Separation Approach

A.1 Fractionation by Ultrafiltration

Settlement percentages of *C. gigas* larval response to fractionated forms of CgSE with varying extract amounts are shown in Figure S1. Fractionation by ultrafiltration was done by separating molecular weight cut-off (MWCO) of the settlement inducing compound into (a) >100K and the <100K fraction was further sub-divided into (b) <50K and (c) >50K CgSE fractions. All fractions except <50K CgSE showed a significant amount of extract-dependent settlement inducing activity ($p < 0.05$). Moreover, quasi-binomial glm analysis showed that at 1 μg, all fractions elicited different larval settlement inducing responses that ranged from 0 to 46%, the highest of which came from >50K CgSE (46%). On the other hand, both crude CgSE and >50K CgSE with settlement percentages of 70% and 74%, respectively, were shown to elicit the highest settlement response at 100 μg amount of the extract ($p < 0.05$). The >50K CgSE was then chosen for the next purification step, Gel filtration chromatography.

A.2 Gel filtration of >50K CgSE

The active fraction, >50K CgSE, was then applied to Superdex 200 10/300 GL column and eluted with 0.15 M NaCl, using the FPLC system. Most of the proteins were eluted in the high molecular mass fraction (F2) while some proteins were of low molecular mass (F3 to F6) as shown in Figures S2 and S3. Characterization of proteins that were visualized only by Stains-all following SDS-PAGE is found in Figure S3A. This cationic dye binds to anionic sites within Ca2+ -binding proteins and stains dark blue or purple for highly acidic and phosphorylated proteins [56]. While proteins of other classes stain red or pink [56]. Stains-all has also been widely used to identify proteins that contain high levels of sulfated sugar residues, acidic proteins, and phosphoproteins in mineralized tissues such as those derived from shell, tooth, and bone [56,79]. In Figure S3A, CgSE showed three prominent, blue-stained bands at 42, 44, and 48 kDa. While gel filtration eluted fraction F2 also showed 3 closely associated bands similar to CgSE at 42, 45, and 50 kDa, but with some 'polydisperse' smeared background around these positions. However, fraction F3 did not show any protein bands suggesting that this active fraction might be composed of low molecular weight proteins. Hence, this additional factor was considered in favor of fraction F2 which was chosen for the next purification step. In terms of the larval settlement bioassay results and as shown in Figure S3 B, no significant response was elicited at the void volume in all extract amounts (fraction, F1; $p > 0.05$). Fraction, F2, with a molecular mass range of 41 to 118 kDa, showed a consistent high settlement inducing activity of 7%, 46%, and 52% at 1, 10, and 50 μg, respectively, and in comparison, to other eluted fractions ($p < 0.05$). It is interesting to note, that while F2 showed a consistent settlement inducing effect on the oyster larvae across all extract amounts, low molecular mass fractions F3, F4, and F5 elicited high inducing effect at 50 μg with settlement percentages at 55%, 55%, and 28%, respectively ($p < 0.05$).

A.3 Separation of F2 proteins

To determine whether the polypeptides in the active fraction F2 could elicit a settlement inducing response in the oyster larvae, each polypeptide was isolated and subjected to a bioassay. The fraction, F2, was resolved in SDS-PAGE under nonreducing and reducing (in the presence of 2-mercaptoethanol) [79] conditions (Figure S4A). At a high amount of sample (60 μg) loaded on each lane and under reducing conditions, only 3 prominent bands appeared at positions 45, 48, and 53 kDa. While under non-reducing conditions, more bands were observed such that, at positions 35, 38, 43, 48, and 60 kDa, the authors were able to isolate these polypeptides. However, among these polypeptides, bands 43 and 48 kDa showed the most dominant signal. Bioassay results of active fraction F2 and its isolated polypeptides are found in Figure S4B. Settlement inducing activity was not observed in all isolated polypeptides under reducing conditions. This suggests that disulfide bonds might be necessary for the folding and stabilization of the settlement inducing factors in this cue [44]. However, under nonreducing conditions, II, IV, and V polypeptides of active fraction F2 showed significant induced

larval responses compared with the control where larvae were immersed on the multi-well with filtered seawater only (C-FSW) ($p < 0.05$). Notably, the settlement percentages of the isolated polypeptides were lower compared to the active fraction (F2) contrary to other reported gregarious cues in other marine invertebrates when the usual effect of a purified cue was stronger [46]. Thus, these results imply that these isolated polypeptides may need to interact synergistically to create a strong and stable inducing signal as a group. Amino acid sequence analysis using a protein sequencer failed to identify these active polypeptide components most likely due to their *N*-termini being blocked.

B. Molecular characterization of Gigasin-6 isoform X1

B.1 Bioinformatic characterization of Gigasin-6 isoform X1 protein sequence

As shown in Supplemental Figure S6, Gigasin-6 isoform X1 has a theoretical molecular mass of 64 kDa (563-aa-long residue) while isoform X2 is a 63 kDa (554-aa-long residue) protein. The disparity of molecular masses between the observed and theoretical mass may be due to the additional presence of post-translational modifications [99]. Gigasin-6 isoform X1 was chosen for further protein sequence characterization and qRT-PCR analyses due to its complete and longer length of amino acid residues that best represents the Gigasin-6 family of proteins. Removal of the first 33 amino acid signal peptide sequence results in a 530 amino acid protein with a calculated molecular weight of 59,920 Da. The amino acid composition of the mature form of this protein is rich in Leu (9.8%), Thr (9.1%), and Asp (8.1%). It has 71 negatively charged residues (Asp + Glu) and 65 positively charged residues, with a theoretical pI of 5.74. A SMART search for protein domains suggests that Gigasin-6 isoform X1 contains a Beta-lactamase domain but may be an inactive form of lactamase-related protein [57,100]. While InterProscan predicts this protein with a non-cytoplasmic domain and contains a transmembrane helix. However, further analysis by the Transmembrane Helices Hidden Markov Model (TMHHH) on the amino acid residues at positions 17 to 36, which was predicted as a transmembrane helix site, revealed that this site coincides within the signal peptide and was not considered to be a true transmembrane helix. Low Complexity Regions (LCR) were identified using IUPRED search and are illustrated in Figure 5A, Supplemental Figure S6. Three LCR sites were shown to be distributed throughout the entire protein sequence. In this study, the mass spectrometry analysis identified the majority of the peptide sequences except those within the LCR and close to the signal peptide region. To determine post-translational modifications, Net*N*Glyc search predicted 5 putative *N*-glycosylation sites, of which two of these sites were identified by mass spectrometry analysis in this study. Other *N*-glycosylation sites were found within low complexity regions which do not contain any tryptic cleavage sites. Using another kind of enzyme or a combination of enzymes might enable mass spectrometry identification for these remaining *N*-glycosylation sites. Net*O*Glyc search suggests 12 possible *O*-glycosylation sites. Use of NetPhos search predicted 70 phosphorylation sites while motif analyses by ScanProsite suggested 24 out of the 70 identified phosphorylation sites might be related to signal transduction processes: 7 Protein Kinase C, 15 Casein Kinase II, 1 Tyrosine, and 1 cAMP and cGMP dependent protein kinase phosphorylation sites. Also, ScanProsite predicted 1 RGD cell attachment sequence at positions 177 to 179.

B.2 Localization of N-glycosylation sites by Mass spectrometry analysis

N-glycosylation sites on Gigasin-6 isoform X1 and/or X2 were identified by comparing mass fingerprint patterns of the glycosylated and deglycosylated bands B5 and B7, respectively (Figure S8). Treatment of glycoproteins with PNGase F causes a 0.98 Da mass shift at the *N*-glycosylation site when this reaction converts *N*-linked asparagine residues into aspartic acid residues [58]. The deamination of an occupied asparagine residue by PNGase F combined with mass determination by MALDI MS is often sufficient to assign *N*-glycosylation sites [83]. Through this strategy, 4 newly appeared m/z signals were identified at 2397.12, 2525.21, 2238.23, and 2254.19 (Figure S8B and Supplemental Table 2). Figures S8C and D present zoomed regions of the MALDI MS/MS fragmentation spectra of the putative *N*-glycosylation sites in the deglycosylated digest. The first two of these signals (Figure S8C) were identified through an error-tolerant search while the latter two signals (Figure S8D) by manual

annotation of the reported spectra with the aid of the MS-Product bioinformatics tool. The peptide with an m/z signal of 2397.12 corresponds to the position of Asn521 carrying the putative *N*-linked (NST) site (^{521}**<u>NST</u>**YIEAFTVDKFDDAKFER^{540}). CID fragmentation spectrum of this ion shows intense signals at fragment ions y_5,y_8, y_9, y_{14}, and b_{19}, corresponding to the PNGase-F-generated aspartic acids of the peptide (Figure S8C). The second m/z 2525.21 signal which showed the highest peak intensity was predicted to result from the same peptide (Figure S8C). CID fragmentation spectrum of this ion shows intense signals at fragment ions y_5, y_6, y_9, y_{14}, b_{15}, and b_{19}. Also, Mascot search results indicate that this signal came from the protonated parent ion that gained a Glu (+ 129 Da) residue giving it an overall m/z shift from 2397.12 to 2525.21. Hence, using an error-tolerant search, this fragment ion was predicted as an actual modification on this peptide containing an *N*-glycosylation site. Interestingly, m/z 2525.21, has not been recorded in any current protein database. Therefore, this is the first time to report the existence of this actual m/z signal for this protein. This may be part of the actual post-translational modification of this protein. Moreover, the third and fourth signal at m/z 2238.23 and 2254.19 corresponds to the position of Asn298 carrying the putative *N*-linked (NGS) site from the same peptide (^{291}FMNYLLG**<u>NGS</u>**IPGTNDVLLAK^{309}) with the expected mass shift following the glycan moiety removal. Although the CID fragmentation pattern of these ion signals (Figure S8D) was of low intensity to yield sufficient confirmation of its sequence through error tolerant search, their fragment ion y and b series patterns were consistent with the predicted fragment ion profiles of this sequence in MS-Product bioinformatic tool as shown in Figure S8D. Using a different endopeptidase with a combination of trypsin with another endopeptidase might yield a stronger signal intensity for this putative *N*-glycosylation site. However, the presence of these m/z values and the positive unitary mass shift that occurred after PNGase F treatment, combined with the absence of the same m/z value in the glycosylated sample, strongly support the assignment made [58,83].

C. Molecular characterization of Stains-all stainable acidic proteins

By exploring the putative identity of the dominant and highly acidic, phosphorylated 48 kDa band, its biochemical properties were compared to other identified highly acidic and phosphorylated shell matrix proteins among the *Crassostrea* species. The 48 kDa band, which was demonstrated in this study to exhibit a settlement inducing activity, was putatively identified as a homolog of folian-cv1 from *Crassostrea virginica* based on their commonly observed biochemical characteristics [40]. Among these characteristics include (1) both were extracted from the EDTA-soluble shell matrix; (2) both are Asp-N rich, which is a common component reported in EDTA-soluble organic matrices [40,13]; (3) band position was also reported at 48 kDa in folian cv-1; (4) Folian cv-1 is moderately glycosylated and highly phosphorylated while the 48 kDa protein in this study did not show any detected glycosylation after Pro-Q Emerald staining or nor any observed band shift after PNGase F deglycosylation treatment (Figure 2). But it showed that just like folian cv-1, it was highly phosphorylated as demonstrated by the intense band signal following Pro-Q Diamond phosphoprotein staining as well as Stains-all staining methods (Figures 3, 4, and Figures S3, S4, respectively); (5) 48 kDa band in this study was endoprotease-resistant with trypsin, chymotrypsin, Glu-C which suggests that it may have an amino acid composition similar to those reported in folian cv-1; (6) Other shell organic matrix extracted along folian cv-1 showed common epitopes suggesting they are related [40]. Mass spectrometry analysis of the stains-all stainable acidic proteins in this study also shared similar m/z signal chemical signatures suggesting that they may contain similar chemical structures. Hence, a blast search from folian cv-1 and a multiple sequence alignment was performed on these homologs as shown in Supplementary Figure S9.

Results of this analysis revealed dentin sialophosphoprotein-like in *C. gigas* has a 95.83% homology with Folian cv-1 (E-value = 0.006). Dentin sialophosphoprotein-like is a protein with 331-aa-long residue. Removal of the 18 amino acid signal peptide sequence reveals a mature protein with 313 amino acid residues and a calculated molecular weight of 31,992 Da as well as a theoretical pI of 2.26. It is predicted to be rich in Asp (36%), Ser (43.1%), and Glu (7.7%). It has 136 negatively charged residues (Asp + Glu), and only 1 positively charged residue (Arg + Lys). ScanProsite profile suggests that it is also Asp- and Ser-rich. There were 149 putative phosphorylation sites and 4 *N*-myristoylation sites

predicted. No putative *N*-glycosylation site was found in this protein sequence. This result agrees with the PNGase F deglycosylation result in this study wherein no band shift was observed after this treatment. However, Net*O*Glyc predicted 137 putative *O*-glycosylation sites. Likewise, this predicted amino acid composition also confirms the endoproteinase-resistant results in this study which shows susceptibility for missed or lack of cleavage sites: 1 Arg residue; absence of Lys, Phe, Trp, Leu; 10 Tyr and 24 Glu residues. The latter two residue types might be sandwiched by amino acid residues that could yield a missed cleavage as previously discussed in this study. Prediction of intrinsically unstructured proteins and detection of low complexity regions (LCRs) were done using IUPred3 software. IUPred3 analysis of this protein sequence revealed that Dentin sialophosphoprotein-like is considered a putative intrinsically disordered protein. To date, the functional role of this protein in *C. gigas* is unclear and its sequence was predicted only by automated computational analysis derived from a genomic sequence [60]. Nonetheless, even though CGDSP has high sequence homology to folian cv-1, it contains some amino acid residues, sequence insertions, and deletions that are unique to itself (Supplemental Figure S9).

D. Molecular characterization of Surface protein P12p-like

Peptide Mass Fingerprinting of the 17 kDa band was identified as the product(s) of two isoform genes of Surface protein P12p-like (CGS12P) (accession numbers: XP_034319257.1 and XP_034321529.1) as shown in Table 1. In Figure S10A, a sequence alignment comparison of these two variants indicates they have identical amino acid compositions except for one substitution of an amino acid residue at position 7, where a Phe is replaced by Leu. These two isoform genes of Surface P12p-like in *C. gigas* are a 14,982 and 15,016 Da protein containing 128 amino acid residues. The difference between its observed and theoretical molecular mass could be attributed to the additional presence of post-translational modifications in this protein [99]. Removal of the first 20 amino acid peptide signal sequence results in a 108 amino acid protein with a calculated molecular weight of 12,279.91 Da. Its amino acid composition is rich in Asp (15.7%), Asn (13.9%), Gly (8.3%), and Glu (7.4%). It also has 25 negatively charged amino acid residues (Asp + Glu) and 13 positively charged residues. (Arg + Lys) and with a theoretical pI of 4.30. InterProscan search revealed that except for its signal peptide region, the entire sequence length of this protein contains a non-cytoplasmic domain, indicating that it may be located in the extracellular region. Half of the sequence length from the N-terminal region (position 23-73) was predicted to be an intrinsically disordered region or low complexity region (LCR). Notably, the LCR region is 28% Asn-, 23% Asp-, 17% Gly- rich and with several post-translational modifications (Figures 6A and 7). Mass spectrometry analysis in this study could identify half of the entire sequence length towards the C-terminus but was unable to identify the location of the actual N-glycosylation modified sites and their corresponding peptide sequence due to the lack of tryptic cleavage sites on the N-terminal region. This protein showed a low-intensity band pattern in SDS-PAGE following Pro-Q Emerald (Figure 2) and Pro-Q Diamond staining (Figure 3) for glycoprotein and phosphoprotein, respectively. These results match with the predicted low-level yet diverse post-translational modifications on this protein. It contains 1 predicted *N*-glycosylation and 6 *O*-glycosylation sites, 2 *N*-myristoylation sites, 4 Casein kinase II phosphorylation sites (at positions 52-55, 53-56, 85-88, 87-90), 3 Protein kinase C phosphorylation site (at positions 78-80, 87-89, 118-120), and 5 other phosphorylation sites.

To compare the CGS12P with its homologs, a multiple sequence alignment was performed. Figure S11 shows CGS12P to have homology with other proteins specific to *Crassostrea gigas*, but no homology was found in other organisms. The functional role of this gene in these organisms is still unclear. On the other hand, CGS12P was closely related to a pheromone-processing carboxypeptidase KEX1-like in *Crassostrea gigas* by 85% homology identity (E-value = 9×10^{-119}, NCBI Blastn). However, little is known about the functional significance of KEX1 in *C. gigas*. Notably, this is the first report implicating the actual presence of this protein as part of the adult shell matrix in *C. gigas.* In addition, this protein sequence that was archived in the NCBI database was predicted only by automated computational analysis derived from a genomic sequence [60].

Supplementary figures

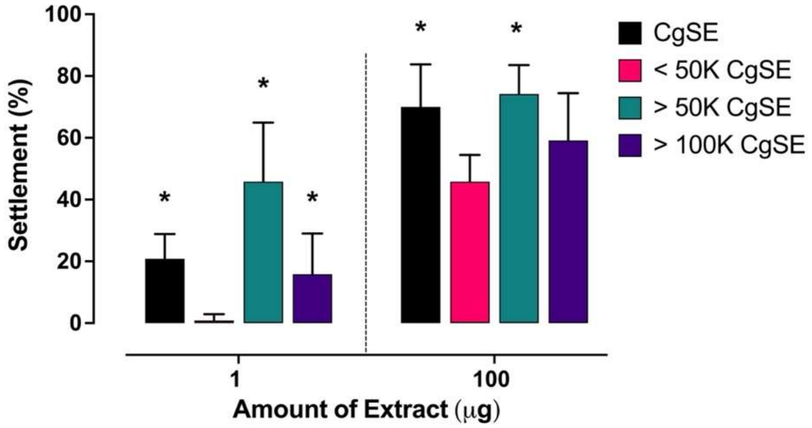

Figure S1. Settlement percentages of *C. gigas* larvae on different ultrafiltration fractions with varying amounts of CgSE after 24 h. Asterisks (*) denote significant differences in the amount coated from different fractionated forms of CgSE by ultrafiltration, determined via quasi-binomial glm ($p < 0.05$, n = 6, using different batches of larvae).

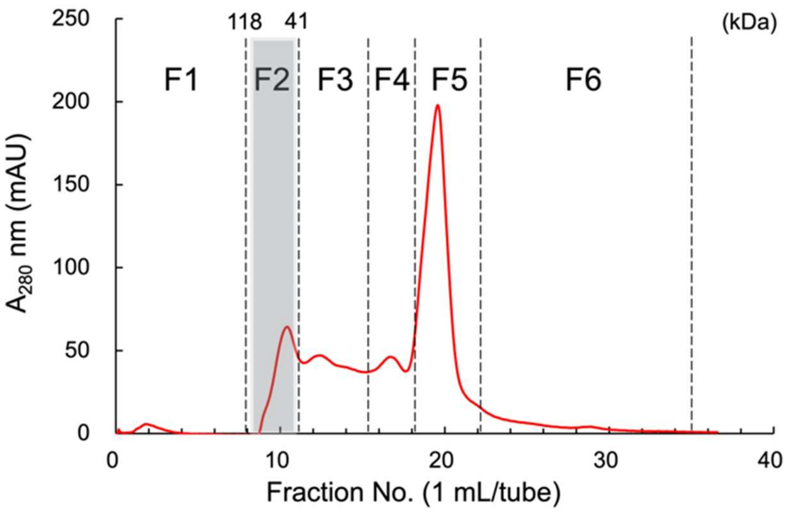

Figure S2. Chromatogram profile of fractions eluted from >50K CgSE gel filtration chromatography. Abbreviations: F1 to F6 = eluted pooled fractions.

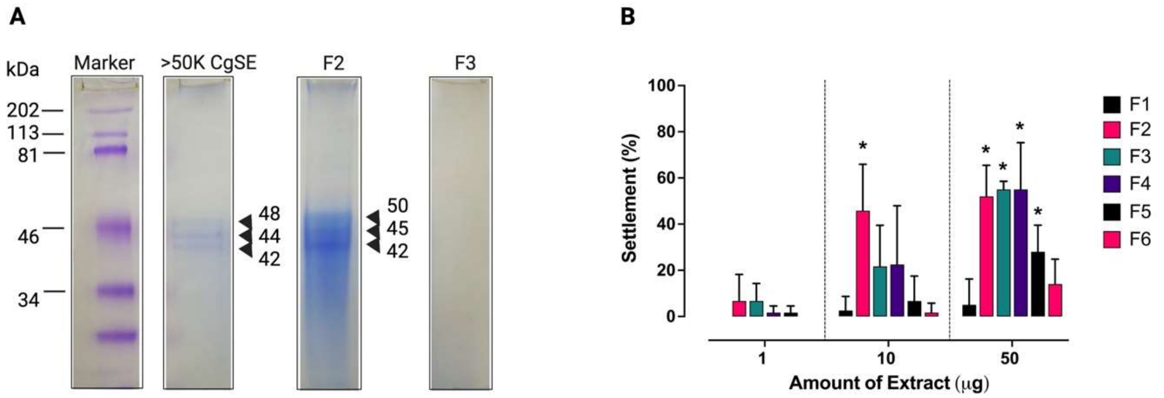

Figure S3. SDS-PAGE analysis and larval settlement bioassay after >50K CgSE Gel filtration chromatography. (A) Ten μg each of the active fractions >50K CgSE, F2, and F3 were resolved on a 10% polyacrylamide gel under reducing condition and stained with Stains-all. (B) Settlement Percentages of *C. gigas* larvae on different pooled fractions from >50K CgSE gel filtration chromatography with varying amounts of extract after 24 h. Asterisks (*) denote significantly settlement inducing groups, determined via quasi-binomial glm ($p < 0.05$).

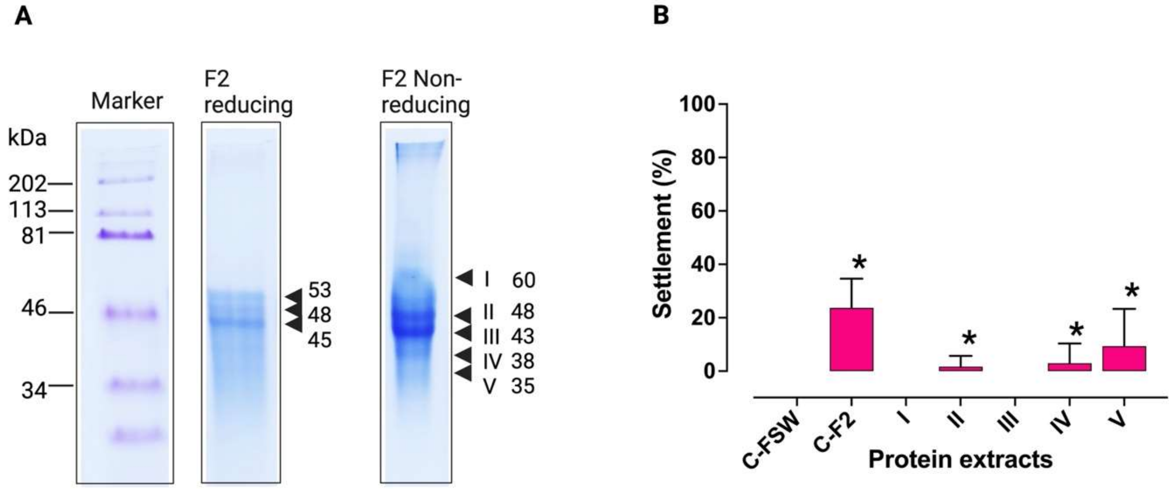

Figure S4. SDS-PAGE profile and larval settlement bioassay of active fraction F2 and its isolated polypeptides. (**A**) Sixty µg of active fraction F2 was treated under reducing and nonreducing conditions and resolved on a 10% polyacrylamide gel. (**B**) Settlement percentages of *C. gigas* larvae on different isolated polypeptides from active fraction F2 under a nonreducing condition with varying amounts of extract after 24 h. Asterisks (*) denote significant differences in larval response to the inducing cues, using C-FSW as the baseline, determined via quasi-binomial glm (p < 0.05, n = 6).

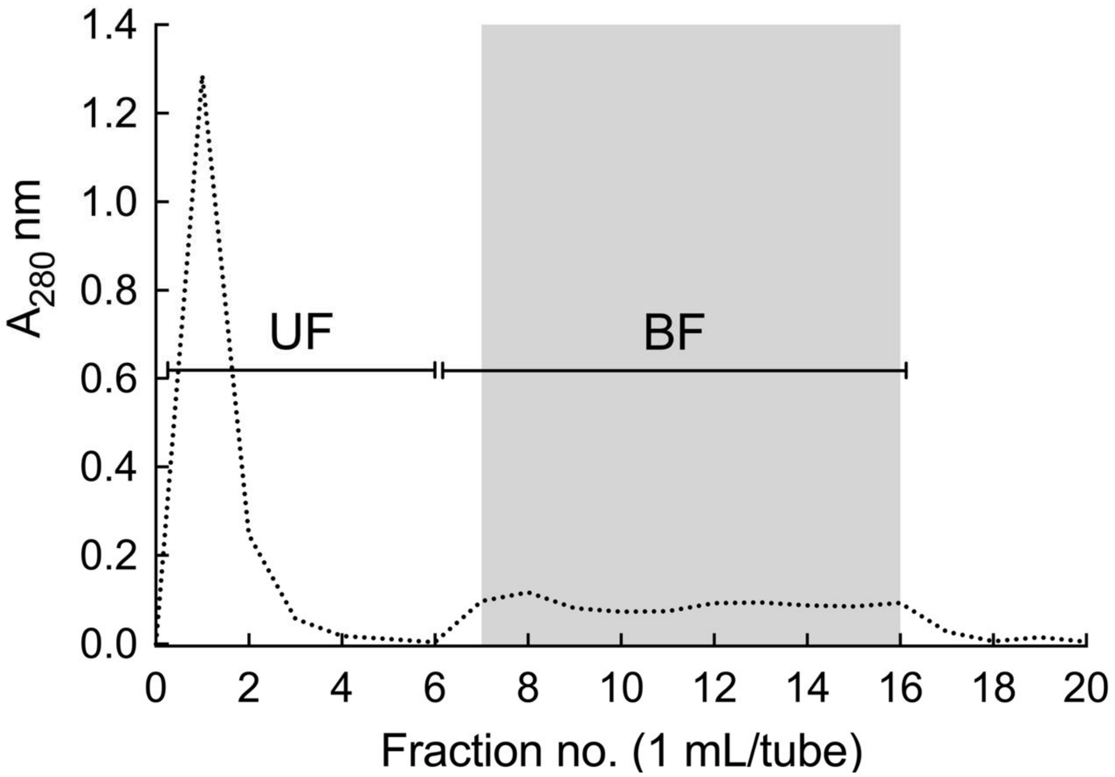

Figure S5. Chromatogram profile of fractions eluted after WGA affinity chromatography under 1.0 M NaCl buffer system. Abbreviations: UF = Unbound fraction component; BF = Bound fraction component; BS = Buffer System.

```
XP_011449647.2_G6iX1 MNNTQGASFVKMSSRNLLYSSVVLFLVLFYCHGGPLEDRVRSTIQEVYKNCRKDKNPGVI 60
XP_011449648.2_G6iX2 MNNTQGASFVKMSSRNLLYSSVVLFLVLFYCHGGPLEDRVRSTIQEVYKNCRKDKNPGVI 60
P86789.1_G6          -----------MSSRNLLYSSVVLFLVLFYCHGGPLEDRVRSTIQEVYKNCRKDKNPGVI 49
                                ************************************************

XP_011449647.2_G6iX1 VSVVKDGQNVLTEALGVKDKISGEAITTDTLFGLGGISALFANILIAKKNAEYADSNNPD 120
XP_011449648.2_G6iX2 VSVVKDGQNVLTEALGVKDKISGEAITTDTLFGLGGISALFANILIAKKNAEYA------ 114
P86789.1_G6          VSVVKDGQNVLTEALGVKDKISGEAITTDTLFGLGGISALFANILIAKKNAEYA------ 103
                     ******************************************************

XP_011449647.2_G6iX1 STLEMDEDTTLRNLFGNNKLFEKSKLRSRYATSLDVMAHRLGFKNTPYLFLDDTVTRGDP 180
XP_011449648.2_G6iX2 ---EMDEDTTLRNLFGNNKLFEKSKLRSRYATSLDVMAHRLGFKNTPYLFLDDTVTRGDP 171
P86789.1_G6          ---EMDEDTTLRNLFGNNKLFEKSKLRSRYATSLDVMAHRLGFKNTPHLFLDDTVTRGDP 160
                        ********************************************:************

XP_011449647.2_G6iX1 VIQRISSMKPRGRFRDSFYYNELMYSILTTIGERLGRDSWENLVKNEIYTPLGMAKSKFF 240
XP_011449648.2_G6iX2 VIQRISSMKPRGRFRDSFYYNELMYSILTTIGERLGRDSWENLVKNEIYTPLGMAKSKFF 231
P86789.1_G6          VIQRISSMKPRGRFRDSFYYNELTYSILTTIGERLGRDSWENLVKNEIYTPLGMAKSKFF 220
                     *********************** ************************************

XP_011449647.2_G6iX1 TTLDPSTVDIARAYKEDDGSLFPVPFEFLKKWSSLCSTTCVLSSANDMSKFMNYLLGNGS 300
XP_011449648.2_G6iX2 TTLDPSTVDIARAYKEDDGSLFPVPFEFLKKWSSLCSTTCVLSSANDMSKFMNYLLGNGS 291
P86789.1_G6          TTLDPSTVDIARAYKEDDGSLFPVPFEFLKKWSSLCSTTCVLSSANDMSKFMNYLLGQRK 280
                     ********************************************************: .

XP_011449647.2_G6iX1 IPGTNDVLLA-KKIHRDLFDAYNRLQDPSIEDYFLTIRGVPVSRTHFAYAMGIKRGMYNN 359
XP_011449648.2_G6iX2 IPGTNDVLLA-KKIHRDLFDAYNRLQDPSIEDYFLTIRGVPVSRTHFAYAMGIKRGMYNN 350
P86789.1_G6          PSWTKPCVTGPRKFTLILFDAI-------------------------------------- 302
                        *: : .  :*:   ****

XP_011449647.2_G6iX1 ERILETADDMHGYNTLMTLFPDRNLGIFIAMTGEDKKDLFRTALSSYISDLYLDKEPWLN 419
XP_011449648.2_G6iX2 ERILETADDMHGYNTLMTLFPDRNLGIFIAMTGEDKKDLFRTALSSYISDLYLDKEPWLN 410
P86789.1_G6          ------------------------------------------------------------ 302

XP_011449647.2_G6iX1 SSLLCTFPEPFMKGPDPDTPKVHPVVPLGRLPTDFTGTYTNDIYGKMEIIDKSGVLEAKY 479
XP_011449648.2_G6iX2 SSLLCTFPEPFMKGPDPDTPKVHPVVPLGRLPTDFTGTYTNDIYGKMEIIDKSGVLEAKY 470
P86789.1_G6          ------------------------------------------------------------ 302

XP_011449647.2_G6iX1 GYATFDLQREETTSLKFNMFPTGLIRHMFSVDDLRFREMKNSTYIEAFTVDKFDDAKFER 539
XP_011449648.2_G6iX2 GYATFDLQREETTSLKFNMFPTGLIRHMFSVDDLRFREMKNSTYIEAFTVDKFDDAKFER 530
G6                   ------------------------------------------------------------ 302

XP_011449647.2_G6iX1 VQPVTNTTTTTTTEKPAGGIAIPL 563
XP_011449648.2_G6iX2 VQPVTNTTTTTTTEKPAGGIAIPL 554
P86789.1_G6          ------------------------ 302
```

Figure S6. Multiple sequence alignment of Gigasin-6 and its isoforms. The predicted signal peptide (aa 1-33) is printed blue. Predicted *N*-glycosylation sites are printed in red while those enclosed in red boxes indicate identified actual *N*-glycosylation sites. Peptides sequenced by MS/MS containing the observed *N*-glycosylation sites are underlined. Predicted *O*-glycosylation sites are enclosed in black boxes. Potential Phosphorylation sites are shaded green while *N*-myristoylation sites are shaded blue. Cysteine-containing residues are shaded yellow while the cell attachment-containing sequence, RGD, is shaded pink. Gigasin 6-isoform X1 shows an extra and unique residue sequence printed in purple. Low complexity regions are doubly underlined and consist of T-rich (37%) residues. Dashes denote blanks or gaps. Asterisks, colons, and dots represent identical amino acids, conserved substitutions, and semi-conserved substitutions, respectively.

```
XP_011449647.2_Cg_G6iX1  MNNTQGASFVKMSSRNLLYSSVVLFLVLFYCHGGPLEDRVRSTIQEVYKNCRKDKNPGVI  60
XP_021348470.1_My_UP     -----------MEVQRI-LGFFFLTLIFSLAESGLREEEADKTLRSVL---ACNQNPGMA  45
XP_033750192.1_Pm_pfl    -----------MERQRM-ITFYYLLLVFSLAESGLKEEEVDKTLRMVL---ACNKNPGMA  45
XP_048735123.1_Oe_G6l    -----------MSTRNVLCSLFVLCCVVFTCH-GTIEDEIRTTIQNAYSDCRHYRNPGMV  48
XP_022334982.1_Cv_G6liX1 -----------MSSRTLLSSLFVSCLVLLTCY-GQVEDQIRATLTSVYANCRNQKNPGLI  48
                                    *. : :         :.  .  *  *:.   *:  .       :***:

XP_011449647.2_Cg_G6iX1  VSVVKDGQNVLTEALGVKDKISGEAITTDTLFGLGGISALFANILIAKKNAEYADSNNPD  120
XP_021348470.1_My_UP     VSVVKDGHIVFSKGYGVKNLETKEPVTNKTLFGIASLSKAFASTLLVKLLHR--------  97
XP_033750192.1_Pm_pfl    ISVVKDGRIVFSKGYGVKNLETKEPVSNKTIFGIASLSKAFASTLLVKLLHR--------  97
XP_048735123.1_Oe_G6l    VSVVKDGQNVFSEAYGVKDQISQEPVTTDTLFGLGGISALFANILIAKKSAEYTEY----  104
XP_022334982.1_Cv_G6liX1 VSVVKDGQAVLTEAYGVKDKISSEPVTTDTLFGLGGLSALFANMLIAKKNQDYADSNNAE  108
                         :******: *:::. ***:  : * ::..*:**:..:*  **. *:.*

XP_011449647.2_Cg_G6iX1  S--TLEMDEDTTLRNLFGNNKLFEKSKLRSRYATSLDVMAHRLGFKNTPYLFLDDTVTRG  178
XP_021348470.1_My_UP     ---TKEITLDTEISKIYNDDNIFS-DDLRSRYVTIRDLLAHNIGIRSNNYMRFDDTLTRG  153
XP_033750192.1_Pm_pfl    ---TKEITLDTEISRIYNDDNIFS-DDLRSRYVTIRDLLAHNIGIRSNNYMRFDDSLTRG  153
XP_048735123.1_Oe_G6l    -----NIDEDITLKRLFGNDTLFRKSKLRSRYATTLDIMSHRLGIKNTPYLFLDDSIDRG  159
XP_022334982.1_Cv_G6liX1 NQVTESMDEDTTLRNLFGNNKLFDKSKLRSRYATSLDIMAHRLGFKNTPYLLLDDTVSRG  168
                              .:  *  : .::.::.:*  ..*****.*  *:::*.:*::.. *: :**:: **

XP_011449647.2_Cg_G6iX1  DPVIQRISSMKPRGRFRDSFYYNELMYSILTTIGERLGRDSWENLVKNEIYTPLGMAKSK  238
XP_021348470.1_My_UP     N-IYRRIKYLRGRGRFRESFYYSNLMYGVITDIAERLGGKSWEDLVREELLNPIGMMSTT  212
XP_033750192.1_Pm_pfl    N-IYRRIKFLRGRGRFRESFYYSNLMYGIITDIAERLGGKSWEDLVREELLNPIGMSSTT  212
XP_048735123.1_Oe_G6l    DPALHRIKHMKPKGRFRDSFYYNEFVSSILTTIGERLGADSWENLIENEIFYPLGMSNSE  219
XP_022334982.1_Cv_G6liX1 GSILERIAPMKPRGRFRDSFYYNEILYGILTTIGERLGRNGWEELVKDEIYTPLGMTKSG  228
                         .   .**  :: :****:****.::: .::* *.**** ..**:*:.:*:  *:** .:

XP_011449647.2_Cg_G6iX1  FFTTLDPSTVDIARAYKEDDGSLFPVPFEFLKKWSSLCSTTCVLSSANDMSKFMNYLLGN  298
XP_021348470.1_My_UP     FATVAEEKKIDLATGYIDFYGDIHPVSFRLSKVWGNLCGSGCVMSSANDMAKWMMFHLDK  272
XP_033750192.1_Pm_pfl    FATVAEEKKIDLATGYIDFYGEIHPVAFRLSKEWGNLCGSGCVMSSANDMAKWMMFHLDK  272
XP_048735123.1_Oe_G6l    FFTTVAPAMVNIAKGYKDDENSLYPVPYEFLKKWTRLCSTSCVISSANDMAKFMNYLLGN  279
XP_022334982.1_Cv_G6liX1 FLTTVTPANVNIAKAYTNDEGSLYPVSFEFLKKWSRLCSTTCIISSANDMAKFMNYLLSN  288
                         * *.     :::* .* :  ..:.** :.: * *  **.: *::******:*:* : *.:

XP_011449647.2_Cg_G6iX1  GSIPGTNDVLLAKKIHRDLFDAYNRLQDPSIEDYFLTIRGVPVSRTHFAYAMGIKRGMYN  358
XP_021348470.1_My_UP     GRNSFSVR-VVDERALSHTHKAHNTIAKSSIFKYFTKP-VVPHTRCQTNYALGWKNGYYR  330
XP_033750192.1_Pm_pfl    GRNSFSVK-VVDERALSHTHKAHNTIAKSSIFKYFTKP-VVPYTHCQTNYALGWKNGYYR  330
XP_048735123.1_Oe_G6l    GTIPGSNETLLNKRDQENLFDAHNRLKDPSVEDYFLAP-KVPVSRTHCSYAMGIKKGMYN  338
XP_022334982.1_Cv_G6liX1 GTIPGTNTELLNRKVHRDLFDAYNRLQDPSIEDYFLSIRGVPVSRTHFAYAMGIKRGMYN  348
                         *    :   :: .:   . ..*:* : . *: .**     ** :: :  **:* *.* *.

XP_011449647.2_Cg_G6iX1  NERILETADDMHGYNTLMTLFPDRNLGIFIAMTGEDKKDLFRTALSSYISDLYLDKEPWL  418
XP_021348470.1_My_UP     GYEILTHSGSTWGYRALVTLFPAMRIGVYTSMTGEDYGYILRTNIHNYLADMYLEETPWL  390
XP_033750192.1_Pm_pfl    GYEILTHSGSTWGYRALITLFPAMRLGIYTSLTGEDYGYILRTNIHNYLADMYLDEKPWL  390
XP_048735123.1_Oe_G6l    NKRILEVADDLHGYNTMMTLFPDYNLGIFIAMTGEDKDDLFRTTLSSYITDMYLGDQTWL  398
XP_022334982.1_Cv_G6liX1 NERILETADDMHGYNTLMTLFPDHKLGIFIAMTGEDKKDLFRTALSSYISDLYLGKEPWL  408
                         . .**  :..  **.:::****  .:*:: ::****   ::** : .*::*:** .  **

XP_011449647.2_Cg_G6iX1  NSSLLCTFPEPFMK-GPDPDTPKVHPVVPLGRLPTDFTGTYTNDIYGKMEIID--KSGVL  475
XP_021348470.1_My_UP     NASTICSFPEPWFRPGKDKPKPSIDKTRELPRNTTYYVGEYENPAYGRMIVAVNGTTGKL  450
XP_033750192.1_Pm_pfl    NGSTICSFPEPWFRPGKEKPKPLIDKTRALPRNTTYYIGEYENPAYGRMIVAVNGTTGNL  450
XP_048735123.1_Oe_G6l    NSTTMCTFPAPFMP-ALTQDTPEVHPEVPLGRLYEEFVGNYTNKIYGTAEIVF--ENGHL  455
XP_022334982.1_Cv_G6liX1 NSSLLCSFPEPFMK-GPDPDTPRTHPEVPLGRAPEDFIGKYTNDIYGTAEIVS--ERGIL  465
                         *.: :*:** *::  .    .*  .    * *    : * * *  **   :      * *

XP_011449647.2_Cg_G6iX1  EAKYGYATFDLQREETTSLKFNMFPTGLIRHMFSVDDLRFREMKNSTYIEAFTVDKFDDA  535
XP_021348470.1_My_UP     IIKYGEVTLGLYPKA-MKDEFHFESLGFAALVLNFGTIKFKMETLSGYFAAFQVTTFDTK  509
XP_033750192.1_Pm_pfl    IIKYGVVTLGLYPKA-LRDEFHFETLGFAAFVLNFGTIKFKMETLSGYFAAFQVTTFDTK  509
XP_048735123.1_Oe_G6l    GLKYGYATFVLKREKTTSLKFNMFPSGLIEHMFSVDDLRFKERKGSSDIESFRVDKFDNA  515
XP_022334982.1_Cv_G6liX1 VFKYGFGTFDLKREESTSLKFNMFPTGLIHHMFSVDDLRFRQKTNSTNIEAFTVDKFDDA  525
                           ***  *: *  :     :*::   *:   ::... ::*:  . *  : :* * .**

XP_011449647.2_Cg_G6iX1  K---FERVQPVTNTTTTTT------T-----------EKPAGGIAIPL------------  563
XP_021348470.1_My_UP     DPPDFQRFMTQNDLPEV-GNPLYVASQRNSANTSHSAELLFGILIIWIINLFVFRKIPTC  568
XP_033750192.1_Pm_pfl    DPPEFQRFMTQNDLPNT-L-YVGVASQRNSANTLQSMDTPLGLLIIWIINLFIFRKIPVC  567
XP_048735123.1_Oe_G6l    E---FEKVLPATTTTSTTTTPLPAAA-----------IQPAGGIAIPYQGQ---------  552
XP_022334982.1_Cv_G6liX1 K---FVRVVPQTTA-SPTA------T-----------EKPVGGIAIPV------------  552
                         .   * :.   .             :               * : *

XP_011449647.2_Cg_G6iX1   --                                                          563
XP_021348470.1_My_UP      RS                                                          570
XP_033750192.1_Pm_pfl     RS                                                          569
XP_048735123.1_Oe_G6l     --                                                          552
XP_022334982.1_Cv_G6liX1  --                                                          552
```

Figure S7. Multiple sequence alignment of Gigasin-6 isoform X1 with homologous sequences from other bivalves. Dashes denote blanks or gaps. Asterisks, colons, and dots represent identical amino acids, conserved substitutions, and semi-conserved substitutions, respectively. Putative *N*-glycosylation sites of G6ix1 are in red font while those from other organisms are highlighted in yellow. Abbreviations: G6iX1, Gigasin-6 isoform X1 from *Crassostrea gigas*; My_UP, Uncharacterized protein LOC021348470.1 from *Mizuhopecten yessoensis*; Pm_pfl, Protein flp-like from *Pecten maximus*; Oe_G6l; Gigasin-6-like from *Ostrea edulis;* Cv_G6liX1, Gigasin-6-like isoform X1 from *Crassostrea virginica.*

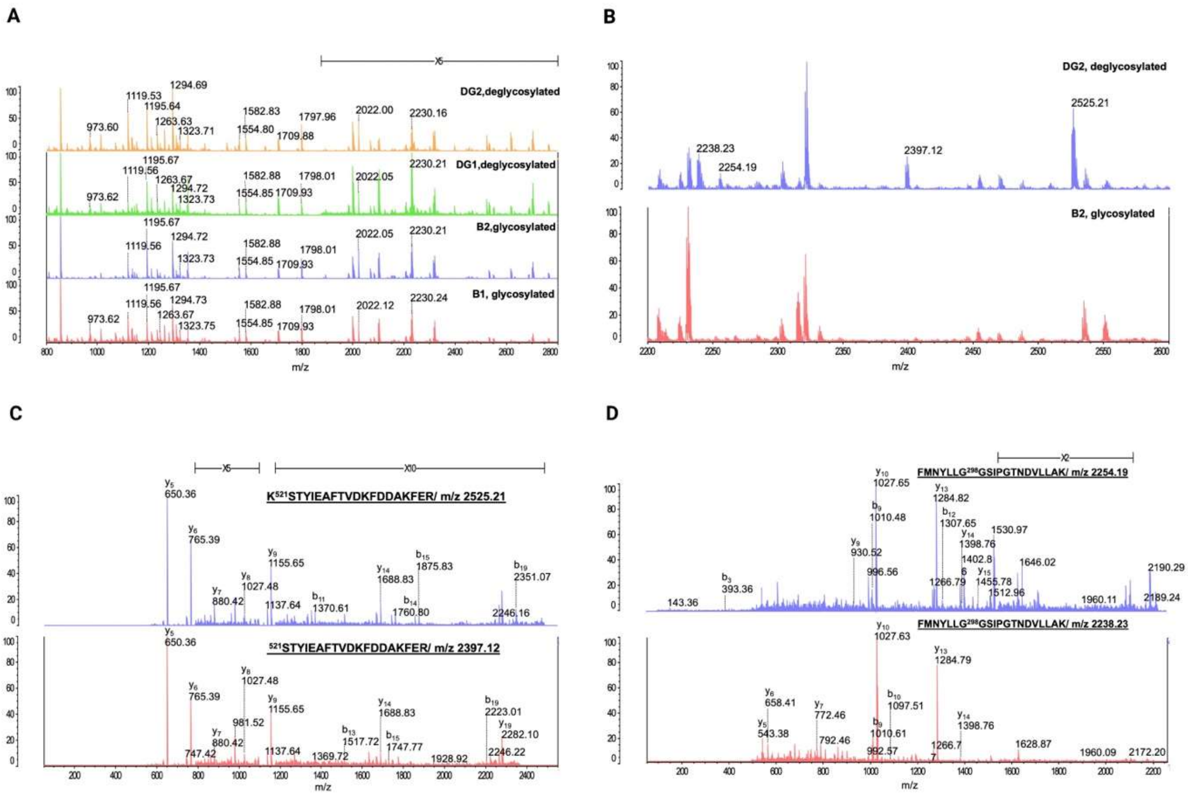

Figure S8. Mass spectrometry analysis on the *Crassostrea gigas* Gigasin-6 isoform X1 and/or X2 and its trimer. (**A**) Mass spectrometric profile of Gigasin-6 isoform X1 and/or X2 and its trimer show similar chemical signatures. (**B**) Localization of *N*-glycosylation sites in Gigasin-6 isoform X1 and/or X2 by PNGase F treatment and mass spectrometry analysis. (**C**) Zoomed region on the MS/MS spectra of identified *N*-glycosylation sites annotated by error-tolerant search. (**D**) Zoomed region on the MS/MS spectra of identified *N*-glycosylation sites by MS-Product bioinformatic tool and manual annotation.

```
XP_034310169.1_CGDSP      MKGLAILVFCALIAVGISYPVPDETTDEPDVTDAPDAGDYDVTD---GPDAYDE------      51
QIB98238.1_Foliancv1,2    MKGLAILVFCALIAVGVSYPVPDEADAGDAYEVADTTDVYDVDTDGYGPDAEEEEEGNGD      60
QIB98237.1_Foliancv1,1    MKGLAILVFCALIAVGVSYPVPDEADAGDAYDVADTTDVYDVDTDGYGPDAEEEEEGNGD      60
XP_022314946.1_CVUP,1     MKGLAILVFCALIAVGVSYPVPDEADAGD---VADTTDVYDVDTDGYGPDAEEEEEGNGE      57
XP_022289736.1_CVUP,2     MKGLAILVFCALIAVGVSYPVPDEADAGD---VADTTDVYDVDTDGYGPDAEEEEEGNGE      57
                          ****************:*******:        *  :. ***    **** :*

XP_034310169.1_CGDSP       ---EGDDDGSDGSESSDSSDSSDSSADDDSDDRSDGESGDDESGD--SDDSDGSDGSDSS      106
QIB98238.1_Foliancv1,2     EEGDGEDDENDDSDSSDSDERDDSSADDDSDDRSDDESGDDEDESDDSIDSEGSDGSDDS      120
QIB98237.1_Foliancv1,1     EEGDGEDDENDDSDSSDSDDSDDSSADDDSDDRSDDESGDDEDESDDSSDSEGSDGSDDS      120
XP_022314946.1_CVUP,1      EEGDGEDDGNDDSDSSDSDDSDDSSADDDSDDRSDDESGDDEDESDDSSDSEGSDASDDS      117
XP_022289736.1_CVUP,2      EEGDGEDDGNDDSDSSDSDDSDDSSADDDSDDRSDDESGDDEDESDDSSDSEGSDASDDS      117
                           :*:** .*.*:****.: .*************.******. .  * **:***.**.*

XP_034310169.1_CGDSP       DSSDSSDSSDSSDSSDSSDSSDSSDSDSYEDDSESDSSESDSGDDDSES-----------      155
QIB98238.1_Foliancv1,2     DDSDD------SDDSDDSDDSDDSDDDSYDDDSESDSESSSSDDDDSDSSSSASSSSSSS      174
QIB98237.1_Foliancv1,1     DDSDD------SDDSDDSDDSDDSDDDSYDDDSESDSESSSSDDDDSDSSSSASSSSSSS      174
XP_022314946.1_CVUP,1      DDSDD------SDDSDDSDDSDDSDDDSYDDDSESDSESSSSDDDDSSSSSSS----SSS      167
XP_022289736.1_CVUP,2      DDSDD------SDDSDDSDDSDDSDDDSYDDDSESDSESSSSDDDDSSSSSSS----SSS      167
                           *.**.      **.**.**.**.**.***:*******..*.*.****.*

XP_034310169.1_CGDSP       DSDSDSESYSDSYSDSSDDDDSESSSDSDSYSDSDSSSDSESESESESESESSSSESGDD      215
QIB98238.1_Foliancv1,2     SSSSESESYSDSYSDEEYSDESD------SYSDSEDSDDSDDSDDD--------------      214
QIB98237.1_Foliancv1,1     SSSSESESYSDSYSDEEYSDESD------SYSDSEDSDDSDDSDDD--------------      214
XP_022314946.1_CVUP,1      SSSSESESDSESYSDEEYSDESD------SSSDSDDSDDSDDSDDS-------DDDDDDD      214
XP_022289736.1_CVUP,2      SSSSESESDSESYSDEEYSDESD------SSSDSDDSDDSDDSDDS-------DD-----      209
                           .*.*:*** *:****.. .*:*:      * ***:.*.**:...:.

XP_034310169.1_CGDSP       DDSDSSDSDSSSSS-SSSSSSSSSDSDSDSSSGSDSDDDSDDDD-SSDSDSDSDSDSDSD      273
QIB98238.1_Foliancv1,2     --DDDDDSDSDSGSDSDSDSDSGSDSDSDSDSGSDSDSDSDDDDDSDDDDSDSDSDSDSD      272
QIB98237.1_Foliancv1,1     --DDDDDSDSDSGSDSDSDSDSGSDSDSDSDSGSDSDSDSDDDDDSDDDDSDSDSDSDSD      272
XP_022314946.1_CVUP,1      DDDDDDDSDSSSGSDSESDSDSDSDSDSDSDSGSDSDSDSDSGSDSDDDDSDSDSDSDSD      274
XP_022289736.1_CVUP,2      --DDDDDSDSSSGSDSESDSDSDSDSDSDSDSGSDSDSDSDSGSDSDDDDSDSDSDSDSD      267
                           .*..****.*.* *.*.*.*.*******.******.***... *.*.***********

XP_034310169.1_CGDSP       SDSYSEEDDDSSDDDDSSDDSYSDEYSDSSDSDSSDSSDSSDNDDSDDDDSSDSSDEN      331
QIB98238.1_Foliancv1,2     SDSDSEEDSDSD---DDDSDDYSEDYSESDSSYSDSESDSD-SDDDDDD-DDDDSDEY      325
QIB98237.1_Foliancv1,1     SDSDSEEDSDSD---DDDSDDYSEDYSESDSSYSDSESDSD-SDDDDDD-DDDDSDEY      325
XP_022314946.1_CVUP,1      SDSNSEEDSNSD---SDDDDSDSEDYSESDGSYSDSESDSD-SDDDDDDDDDDDDSDEY      328
XP_022289736.1_CVUP,2      SDSNSEEDSNSD---SDDDDSDSDDYSESDDSYSYSESDSD-SDDDDDD-DDDDSDEY      320
                           *** ****.:*.   ....*. *::**:*..* *  ..***. .**.***  ..*.***
```

Figure S9. Multiple sequence alignment of NCBI blast searched Folian cv1 homologs. The 48 kDa as the putative CGDSP shows close homology with Folian cv1 variants 1,2. Asterisks, colons, and dots represent identical amino acids, conserved substitutions, and semi-conserved substitutions, respectively. CGDSP, *C. gigas* Dentin sialophosphoprotein-like; Foliancv2, *C. virginica* Folian cv1 variant 2; Foliancv1, *C. virginica* Folian cv1 variant 1; CVUP1, *C. virginica* Uncharacterized Protein LOC111119246; CVUP2, *C. virginica* Uncharacterized Protein LOC111101504.

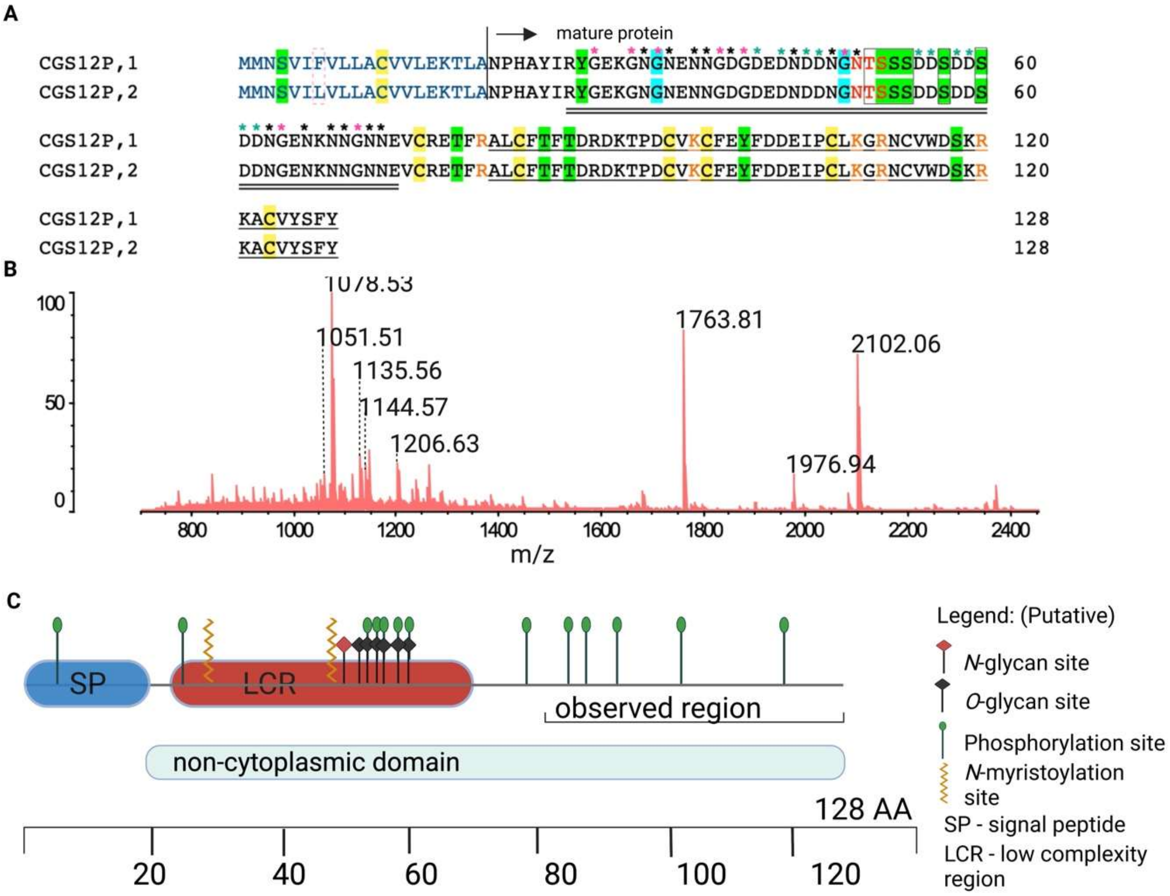

Figure S10. Molecular characterization of *Crassostrea gigas* Surface Protein P12p-like. (**A**) Sequence alignment of Surface Protein P12p-like (CGS12P). Both isoforms contain the same amino acid sequence except for one substituted amino acid position as shown in the enclosed pink box. The Predicted signal peptide (aa 1-20) is in blue. Doubly-lined Low Complexity Region (LCR) domain (aa 23-73) is 28% N-, 23% D-, 17% G- rich and is indicated with * (asterisk) marks. No peptides from the LCR domain which contains *N*- and *O*-glycosylation-rich sites were identified due to a lack of tryptic cleavage sites. Sequences covered by MS spectra are underlined. Tryptic cleavage sites of MS spectra determined sequences are shaded in orange. The predicted *N*-glycosylated sequence is in red while *O*-glycosylation sites are enclosed in black boxes. Predicted phosphorylation sites are shaded green. It also contains several cysteines (yellow-shaded) interspersed along the entire sequence which may be stabilized by disulfide bonds. Predicted *N*-myristoylation sites are shaded in blue. (**B**) Mass spectrometry profile of surface protein P12p-like as represented in the observed region of Figure S10C. (**C**) Graphical representation of Surface protein P12p-like showing protein length, signal peptide (aa 1-20), domains, and post-translation modifications identified by our bioinformatics analysis. The LCR region contains several putative sites of post-translational modifications. Only those peptide fragments close to the C-terminal region were identified by mass spectrometry analysis in this study.

```
XP_034319014.1      MKMQVHVICKSVLHVYQTSSIFFLKTILAGAALFVGCHGYVPPPYPSHSDLTSGSLAAAA 60
XP_034319015.1      ------------------------------------------------------------ 0
XP_034321527.1      ---------------MMNSVILVLLACLVLEKTLANPHAYN-----G------------- 27
XP_034321534.1      ---------------MMNSVILVLLACLVLQKTLANPHAYN-----G------------- 27
XP_034319257.1      ---------------MMNSVIFVLLACVVLEKTLANPHAYI-----R------------- 27
XP_034321529.1      ---------------MMNSVILVLLACVVLEKTLANPHAYI-----R------------- 27


XP_034319014.1      GSIGIVSLAVSAALMSMSSLQNFKDPRKKTTTPEPAESCEDVLAAERQRFEEEKIAEKER 120
XP_034319015.1      --------------MSMSSLQNFKDPRKKTTTPEPAESCEDVLAAERQRFEEEKIAEKER 46
XP_034321527.1      ----------------------YG---EN-DGNENNGDCDVDNGDNENTSSSDDSDDS-- 59
XP_034321534.1      ----------------------YG---EN-DGNENNGDCDVDNGDNENTSSSDDSDDS-- 59
XP_034319257.1      ----------------------YG---EKGNGNENNGDGDEDNDDNGNTSSSDDSDDS-- 60
XP_034321529.1      ----------------------YG---EKGNGNENNGDGDEDNDDNGNTSSSDDSDDS-- 60
                                          :    ::    *   . :     : :  ..:.  :.

XP_034319014.1      FQEEKRDLEARIELSRQIIIGACEEFTAREPIKQPDCIKCFEFLDDQTSCLQA-GSPCAY 179
XP_034319015.1      FQEEKRDLEARIELSRQIIIGACEEFTAREPIKQPDCIKCFEFLDDQTSCLQA-GSPCAY 105
XP_034321527.1      DDREENKEKGNNEVCKNTIRATCDRYTDGD---FEECIECFEYFDDKTQCIQANKRRCLW 116
XP_034321534.1      DDREENKEKGNNEVCKNTIRATCDRYTDGD---FEECIECFEYFDDKTQCIQANKRRCLW 116
XP_034319257.1      DDNGENKNNGNNEVCRETFRALCFTFTDRD--KTPDCVKCFEYFDDEIPCLK--GRNCVW 116
XP_034321529.1      DDNGENKNNGNNEVCRETFRALCFTFTDRD--KTPDCVKCFEYFDDEIPCLK--GRNCVW 116
                     :. :.. :.. *:.:: : . *  :*  :     :*::***::**:  *::     * :

XP_034319014.1      ANFVGVCVYSP---- 190
XP_034319015.1      ANFVGVCVYSP---- 116
XP_034321527.1      DDDRRVCIYDLDIDT 131
XP_034321534.1      DDDRRVCIYDLDIDT 131
XP_034319257.1      DSKRKACVYSFY--- 128
XP_034321529.1      DSKRKACVYSFY--- 128
                     .   .*:*.
```

Figure S11. Multiple sequence alignment of *Crassostrea gigas* Surface Protein P12p-like with its homologs. CGS12P shows homology with other proteins specific to *Crassostrea gigas*, but no homology was found in other organisms. Dashes denote blanks or gaps. Asterisks, colons, and dots represent identical amino acids, conserved substitutions, and semi-conserved substitutions, respectively. Abbreviations: XP_034319014.1, uncharacterized protein LOC117687045 isoform X1 from *Crassostrea gigas*; XP_034319015.1, uncharacterized protein LOC117687045 isoform X2 from *Crassostrea gigas*; XP_034321527.1, pheromone-processing carboxypeptidase KEX1-like from *Crassostrea gigas;* XP_034321534.1, pheromone-processing carboxypeptidase KEX1-like from *Crassostrea gigas;* XP_034319257.1, surface protein P12p-like from *Crassostrea gigas;* XP_034321529.1, surface protein P12p-like from *Crassostrea gigas.*

Supplementary Tables

Table S1. Results of Peptide Mass Fingerprinting (PMF) ion search for protein identification of matrix proteins in the *Crassostrea gigas* EDTA-soluble shell extract.

Band No.	Protein ID	NCBI Accession No.	Sequence	m/z	Delta	Miss	Protein Score	Protein Expect
UB1	Gigasin-6 isoform X2	XP_011449648.2	^{124}NLFGNNKLFEK^{134}	1323.73	0.02	1	79	7.5E-04
			^{141}YATSLDVMAHR^{151}	1263.65	0.04	0	79	7.5E-04
			^{141}YATSLDVMAHR^{151} (Oxidation (M))	1279.65	0.04	0	79	7.5E-04
			^{156}NTPYLFLDDTVTR^{168}	1554.81	0.03	0	79	7.5E-04
			^{228}SKFFTTLDPSTVDIAR^{243}	1797.99	0.05	1	79	7.5E-04
			^{230}FFTTLDPSTVDIAR^{243}	1582.84	0.03	0	79	7.5E-04
			^{307}DLFDAYNR^{314}	1013.51	0.04	0	79	7.5E-04
			^{315}LQDPSIEDYFLTIR^{328}	1709.92	0.05	0	79	7.5E-04
			^{335}THFAYAMGIK^{344}	1138.60	0.03	0	79	7.5E-04
			^{335}THFAYAMGIKR^{345}	1294.71	0.04	1	79	7.5E-04
			^{335}THFAYAMGIKR^{345} (Oxidation (M))	1310.70	0.03	1	79	7.5E-04
			^{346}GMYNNER^{352}	883.40	0.03	0	79	7.5E-04
			^{470}YGYATFDLQR^{479}	1233.62	0.03	0	79	7.5E-04
			^{487}FNMFPTGLIR^{496}	1195.67	0.04	0	79	7.5E-04
			^{487}FNMFPTGLIR^{496} (Oxidation (M))	1211.66	0.04	0	79	7.5E-04
	Gigasin-6 isoform X1	XP_011449647.2	^{133}NLFGNNKLFEK^{143}	1323.73	0.02	1	78	1.2E-03
			^{150}YATSLDVMAHR^{160}	1263.65	0.04	0	78	1.2E-03
			^{150}YATSLDVMAHR^{160} (Oxidation (M))	1279.65	0.04	0	78	1.2E-03
			^{165}NTPYLFLDDTVTR^{177}	1554.81	0.03	0	78	1.2E-03
			^{237}SKFFTTLDPSTVDIAR^{252}	1797.99	0.05	1	78	1.2E-03
			^{239}FFTTLDPSTVDIAR^{252}	1582.84	0.03	0	78	1.2E-03
			^{316}DLFDAYNR^{323}	1013.51	0.04	0	78	1.2E-03
			^{324}LQDPSIEDYFLTIR^{337}	1709.92	0.05	0	78	1.2E-03
			^{344}THFAYAMGIK^{353}	1138.60	0.03	0	78	1.2E-03
			^{344}THFAYAMGIKR^{354}	1294.71	0.04	1	78	1.2E-03
			^{344}THFAYAMGIKR^{354} (Oxidation (M))	1310.70	0.03	1	78	1.2E-03
			^{355}GMYNNER^{361}	883.40	0.03	0	78	1.2E-03

			^{479}YGYATFDLQR^{488}	1233.62	0.03	0	78	1.2E-03
			^{496}FNMFPTGLIR^{505}	1195.67	0.04	0	78	1.2E-03
			^{496}FNMFPTGLIR^{505} (Oxidation (M))	1211.66	0.04	0	78	1.2E-03
UB2	Gigasin-6 isoform X2	XP_011449648.2	^{124}NLFGNNKLFEK^{134}	1323.73	0.02	1	85	2.1E-04
			^{152}LGFKNTPYLFLDDTVTR^{168}	2000.12	0.07	1	85	2.1E-04
			^{156}NTPYLFLDDTVTR^{168}	1554.81	0.03	0	85	2.1E-04
			^{228}SKFFTTLDPSTVDIAR^{243}	1798.01	0.07	1	85	2.1E-04
			^{230}FFTTLDPSTVDIAR^{243}	1582.84	0.03	0	85	2.1E-04
			^{244}AYKEDDGSLFPVPFEFLK^{261}	2102.11	0.06	1	85	2.1E-04
			^{244}AYKEDDGSLFPVPFEFLKK^{262}	2230.21	0.07	2	85	2.1E-04
			^{315}LQDPSIEDYFLTIR^{328}	1709.92	0.05	0	85	2.1E-04
			^{335}THFAYAMGIK^{344}	1138.61	0.04	0	85	2.1E-04
			^{335}THFAYAMGIKR^{345}	1294.71	0.04	1	85	2.1E-04
			^{335}THFAYAMGIKR^{345} (Oxidation (M))	1310.72	0.05	1	85	2.1E-04
			^{470}YGYATFDLQR^{479}	1233.62	0.03	0	85	2.1E-04
			^{487}FNMFPTGLIR^{496}	1195.67	0.04	0	85	2.1E-04
			^{487}FNMFPTGLIR^{496} (Oxidation (M))	1211.65	0.03	0	85	2.1E-04
			^{497}HMFSVDDLR^{505}	1119.56	0.03	0	85	2.1E-04
	Gigasin-6 isoform X1	XP_011449647.2	^{133}NLFGNNKLFEK^{143}	1323.73	0.02	1	84	2.8E-04
			^{161}LGFKNTPYLFLDDTVTR^{177}	2000.12	0.07	1	84	2.8E-04
			^{165}NTPYLFLDDTVTR^{177}	1554.81	0.03	0	84	2.8E-04
			^{237}SKFFTTLDPSTVDIAR^{252}	1798.01	0.07	1	84	2.8E-04
			^{239}FFTTLDPSTVDIAR^{252}	1582.84	0.03	0	84	2.8E-04
			^{253}AYKEDDGSLFPVPFEFLK^{270}	2102.11	0.06	1	84	2.8E-04
			^{253}AYKEDDGSLFPVPFEFLKK^{271}	2230.21	0.07	2	84	2.8E-04
			^{324}LQDPSIEDYFLTIR^{337}	1709.92	0.05	0	84	2.8E-04
			^{344}THFAYAMGIK^{353}	1138.61	0.04	0	84	2.8E-04
			^{344}THFAYAMGIKR^{354}	1294.71	0.04	1	84	2.8E-04
			^{344}THFAYAMGIKR^{354} (Oxidation (M))	1310.72	0.05	1	84	2.8E-04
			^{479}YGYATFDLQR^{488}	1233.62	0.03	0	84	2.8E-04
			^{496}FNMFPTGLIR^{505}	1195.67	0.04	0	84	2.8E-04
			^{496}FNMFPTGLIR^{505} (Oxidation (M))	1211.65	0.03	0	84	2.8E-04
			^{506}HMFSVDDLR^{514}	1119.56	0.03	0	84	2.8E-04

UB3	Surface protein P12p-like	XP_034321529.1	^{81}ALCFTFTDR^{89}	1144.57	0.02	0	80	5.9E-04
			^{81}ALCFTFTDRDKTPDCVK^{97}	2102.06	0.05	2	80	5.9E-04
			^{98}CFEYFDDEIPCLK^{110}	1763.81	0.04	0	80	5.9E-04
			^{98}CFEYFDDEIPCLKGR^{112}	1976.94	0.05	1	80	5.9E-04
			^{111}GRNCVWDSK^{119}	1135.56	0.03	1	80	5.9E-04
			^{113}NCVWDSKR^{120}	1078.53	0.02	1	80	5.9E-04
			^{113}NCVWDSKRK^{121}	1206.63	0.03	2	80	5.9E-04
			^{121}KACVYSFY^{128}	1051.51	0.02	1	80	5.9E-04
	Surface protein P12p-like	XP_034319527.1	Same as “XP_034321529.1”					
B1	Gigasin-6 isoform X1	XP_011449647.2	^{133}NLFGNNKLFEK^{143}	1323.75	0.04	1	97	1.3E-005
			^{150}YATSLDVMAHR^{160}	1263.67	0.05	0	97	1.3E-005
			^{150}YATSLDVMAHR^{160} (Oxidation (M))	1279.65	0.04	0	97	1.3E-005
			^{165}NTPYLFLDDTVTR^{177}	1554.85	0.07	0	97	1.3E-005
			^{237}SKFFTTLDPSTVDIAR^{252}	1798.01	0.07	1	97	1.3E-005
			^{239}FFTTLDPSTVDIAR^{252}	1582.88	0.07	0	97	1.3E-005
			^{316}DLFDAYNR^{323}	1013.51	0.04	0	97	1.3E-005
			^{324}LQDPSIEDYFLTIR^{337}	1709.93	0.05	0	97	1.3E-005
			^{344}THFAYAMGIK^{353}	1138.61	0.04	0	97	1.3E-005
			^{344}THFAYAMGIKR^{354}	1294.73	0.06	1	97	1.3E-005
			^{344}THFAYAMGIKR^{354} (Oxidation (M))	1310.72	0.05	1	97	1.3E-005
			^{441}VHPVVPLGR^{449}	973.62	0.03	0	97	1.3E-005
			^{479}YGYATFDLQR^{488}	1233.63	0.04	0	97	1.3E-005
			^{496}FNMFPTGLIR^{505}	1195.67	0.04	0	97	1.3E-005
			^{496}FNMFPTGLIR^{505} (Oxidation (M))	1211.67	0.05	0	97	1.3E-005
			^{506}HMFSVDDLR^{514}	1119.56	0.03	0	97	1.3E-005
			^{506}HMFSVDDLR^{514} (Oxidation (M))	1135.56	0.04	0	97	1.3E-005
	Gigasin-6 isoform X2	XP_011449648.2	^{124}NLFGNNKLFEK^{134}	1323.75	0.04	1	96	1.5E-005
			^{141}YATSLDVMAHR^{151}	1263.67	0.05	0	96	1.5E-005
			^{141}YATSLDVMAHR^{151} (Oxidation (M))	1279.65	0.04	0	96	1.5E-005
			^{156}NTPYLFLDDTVTR^{168}	1554.85	0.07	0	96	1.5E-005
			^{228}SKFFTTLDPSTVDIAR^{243}	1798.01	0.07	1	96	1.5E-005
			^{230}FFTTLDPSTVDIAR^{243}	1582.84	0.07	0	96	1.5E-005
			^{307}DLFDAYNR^{314}	1013.51	0.04	0	96	1.5E-005

			315LQDPSIEDYFLTIR328	1709.93	0.05	0	96	1.5E-005
			335THFAYAMGIK344	1138.61	0.04	0	96	1.5E-005
			335THFAYAMGIKR345	1294.73	0.06	1	96	1.5E-005
			335THFAYAMGIKR345 (Oxidation (M))	1310.72	0.05	1	96	1.5E-005
			432VHPVVPLGR440	973.62	0.03	0	96	1.5E-005
			470YGYATFDLQR479	1233.63	0.04	0	96	1.5E-005
			487FNMFPTGLIR496	1195.67	0.04	0	96	1.5E-005
			487FNMFPTGLIR496 (Oxidation (M))	1211.66	0.05	0	96	1.5E-005
			497HMFSVDDLR505	1119.56	0.03	0	96	1.5E-005
			497HMFSVDDLR505 (Oxidation (M))	1135.56	0.04	0	96	1.5E-005
B2	Gigasin-6 isoform X1	XP_011449647.2	133NLFGNNKLFEK143	1323.73	0.02	1	63	3.1E-02
			165NTPYLFLDDTVTR177	1554.85	0.07	0	63	3.1E-02
			237SKFFTTLDPSTVDIAR252	1798.01	0.07	1	63	3.1E-02
			239FFTTLDPSTVDIAR252	1582.88	0.07	0	63	3.1E-02
			316DLFDAYNR323	1013.51	0.04	0	63	3.1E-02
			324LQDPSIEDYFLTIR337	1709.93	0.05	0	63	3.1E-02
			344THFAYAMGIK353	1138.61	0.04	0	63	3.1E-02
			344THFAYAMGIKR354	1294.72	0.05	1	63	3.1E-02
			344THFAYAMGIKR354 (Oxidation (M))	1310.72	0.05	1	63	3.1E-02
			479YGYATFDLQR488	1233.63	0.04	0	63	3.1E-02
			496FNMFPTGLIR505	1195.67	0.04	0	63	3.1E-02
			496FNMFPTGLIR505 (Oxidation (M))	1211.66	0.04	0	63	3.1E-02
			506HMFSVDDLR514	1119.56	0.03	0	63	3.1E-02
	Gigasin-6 isoform X2	XP_011449648.2	124NLFGNNKLFEK134	1323.73	0.02	1	63	3.1E-02
			156NTPYLFLDDTVTR168	1554.85	0.07	0	63	3.1E-02
			228SKFFTTLDPSTVDIAR243	1798.01	0.07	1	63	3.1E-02
			230FFTTLDPSTVDIAR243	1582.88	0.07	0	63	3.1E-02
			307DLFDAYNR314	1013.51	0.04	0	63	3.1E-02
			315LQDPSIEDYFLTIR328	1709.93	0.05	0	63	3.1E-02
			335THFAYAMGIK344	1138.61	0.04	0	63	3.1E-02
			335THFAYAMGIKR345	1294.72	0.05	1	63	3.1E-02
			335THFAYAMGIKR345 (Oxidation (M))	1310.72	0.05	1	63	3.1E-02

			^{470}YGYATFDLQR^{479}	1233.63	0.04	0	63	3.1E-02
			^{487}FNMFPTGLIR^{496}	1195.67	0.04	0	63	3.1E-02
			^{487}FNMFPTGLIR^{496} (Oxidation (M))	1211.66	0.04	0	63	3.1E-02
			^{497}HMFSVDDLR^{505}	1119.56	0.03	0	63	3.1E-02
DG1	Gigasin-6 isoform X1	XP_011449647.2	^{133}NLFGNNKLFEK^{143}	1323.73	0.02	1	73	3.4E-03
			^{150}YATSLDVMAHR^{160}	1263.67	0.07	0	73	3.4E-03
			^{165}NTPYLFLDDTVTR^{177}	1554.85	0.07	0	73	3.4E-03
			^{237}SKFFTTLDPSTVDIAR^{252}	1798.01	0.07	1	73	3.4E-03
			^{239}FFTTLDPSTVDIAR^{252}	1582.88	0.07	0	73	3.4E-03
			^{316}DLFDAYNR^{323}	1013.51	0.04	0	73	3.4E-03
			^{324}LQDPSIEDYFLTIR^{337}	1709.93	0.05	0	73	3.4E-03
			^{344}THFAYAMGIKR^{354}	1294.72	0.05	1	73	3.4E-03
			^{344}THFAYAMGIKR^{354} (Oxidation (M))	1310.72	0.05	1	73	3.4E-03
			^{441}VHPVVPLGR^{449}	973.62	0.03	0	73	3.4E-03
			^{479}YGYATFDLQR^{488}	1233.63	0.04	0	73	3.4E-03
			^{496}FNMFPTGLIR^{505}	1195.67	0.04	0	73	3.4E-03
			^{496}FNMFPTGLIR^{505} (Oxidation (M))	1211.68	0.06	0	73	3.4E-03
			^{506}HMFSVDDLR^{514}	1119.56	0.03	0	73	3.4E-03
	Gigasin-6 isoform X2	XP_011449648.2	^{124}NLFGNNKLFEK^{134}	1323.73	0.02	1	72	4.3E-03
			^{141}YATSLDVMAHR^{151}	1263.68	0.07	0	72	4.3E-03
			^{156}NTPYLFLDDTVTR^{168}	1554.85	0.07	0	72	4.3E-03
			^{228}SKFFTTLDPSTVDIAR^{243}	1798.01	0.07	1	72	4.3E-03
			^{230}FFTTLDPSTVDIAR^{243}	1582.84	0.07	0	72	4.3E-03
			^{307}DLFDAYNR^{314}	1013.51	0.04	0	72	4.3E-03
			^{315}LQDPSIEDYFLTIR^{328}	1709.93	0.05	0	72	4.3E-03
			^{335}THFAYAMGIKR^{345}	1294.72	0.05	1	72	4.3E-03
			^{335}THFAYAMGIKR^{345} (Oxidation (M))	1310.72	0.05	1	72	4.3E-03
			^{432}VHPVVPLGR^{440}	973.62	0.03	0	72	4.3E-03
			^{470}YGYATFDLQR^{479}	1233.63	0.04	0	72	4.3E-03
			^{487}FNMFPTGLIR^{496}	1195.67	0.04	0	72	4.3E-03
			^{487}FNMFPTGLIR^{496} (Oxidation (M))	1211.66	0.05	0	72	4.3E-03
			^{497}HMFSVDDLR^{505}	1119.56	0.03	0	72	4.3E-03
DG2	Gigasin-6 isoform X1	XP_011449647.2	^{133}NLFGNNKLFEK^{143}	1323.71	0.00	1	91	5E-005

		^{150}YATSLDVMAHR^{160}	1263.63	0.02	0	91	5E-005
		^{165}NTPYLFLDDTVTR^{177}	1554.80	0.02	0	91	5E-005
		^{237}SKFFTTLDPSTVDIAR^{252}	1797.96	0.02	1	91	5E-005
		^{239}FFTTLDPSTVDIAR^{252}	1582.83	0.02	0	91	5E-005
		^{316}DLFDAYNR^{323}	1013.48	0.01	0	91	5E-005
		^{324}LQDPSIEDYFLTIR^{337}	1709.88	0.01	0	91	5E-005
		^{344}THFAYAMGIK^{353}	1138.57	-0.0	0	91	5E-005
		^{344}THFAYAMGIKR^{354}	1294.69	0.02	1	91	5E-005
		^{344}THFAYAMGIKR^{354} (Oxidation (M))	1310.68	0.01	1	91	5E-005
		^{441}VHPVVPLGR^{449}	973.60	0.01	0	91	5E-005
		^{479}YGYATFDLQR^{488}	1233.59	0.00	0	91	5E-005
		^{496}FNMFPTGLIR^{505}	1195.64	0.01	0	91	5E-005
		^{496}FNMFPTGLIR^{505} (Oxidation (M))	1211.64	0.02	0	91	5E-005
		^{506}HMFSVDDLR^{514}	1119.53	0.00	0	91	5E-005
		^{506}HMFSVDDLR^{514} (Oxidation (M))	1135.52	-0.0	0	91	5E-005
Gigasin-6 isoform X2	XP_011449648.2	^{124}NLFGNNKLFEK^{134}	1323.71	0.00	1	90	5.9E-005
		^{141}YATSLDVMAHR^{151}	1263.63	0.02	0	90	5.9E-005
		^{156}NTPYLFLDDTVTR^{168}	1554.80	0.02	0	90	5.9E-005
		^{228}SKFFTTLDPSTVDIAR^{243}	1797.96	0.02	1	90	5.9E-005
		^{230}FFTTLDPSTVDIAR243	1582.83	0.02	0	90	5.9E-005
		^{307}DLFDAYNR^{314}	1013.48	0.01	0	90	5.9E-005
		^{315}LQDPSIEDYFLTIR^{328}	1709.88	0.01	0	90	5.9E-005
		^{335}THFAYAMGIK^{344}	1138.57	-0.0	0	90	5.9E-005
		^{335}THFAYAMGIKR^{345}	1294.69	0.02	1	90	5.9E-005
		^{335}THFAYAMGIKR^{345} (Oxidation (M))	1310.68	0.01	1	90	5.9E-005
		^{432}VHPVVPLGR^{440}	973.60	0.01	0	90	5.9E-005
		^{470}YGYATFDLQR^{479}	1233.59	0.00	0	90	5.9E-005
		^{487}FNMFPTGLIR^{496}	1195.64	0.01	0	90	5.9E-005
		^{487}FNMFPTGLIR^{496} (Oxidation (M))	1211.64	0.02	0	90	5.9E-005
		^{497}HMFSVDDLR^{505}	1119.53	0.00	0	90	5.9E-005
		^{497}HMFSVDDLR^{505} (Oxidation (M))	1135.52	-0.0	0	90	5.9E-005

Table S2. MS/MS-based identification and characterization of the protein band containing the determined *N*-glycosylation sites of Gigasin-6 isoform X1 and/or X2 in Pacific oyster *Crassostrea gigas*.

Band No.	N-glycosylation Site	Peptide Sequence	Peptide m/z	*y* series ion m/z	*b* series ion m/z	Delta	Miss	Ion Score
Annotation by Error Tolerant Search								
DG2	N521	K[521]STYIEAFTVDKFDDAKFER + [+129.04 at E6]	2525.21	650.36	1370.611	0.0269	2	32
				765.39	1760.80			
				880.42	1875.83			
				1027.48	2351.07			
				1155.58				
				1688.83				
DG2	N521	[521]STYIEAFTVDKFDDAKFER + [+0.98 at N-term N]	2397.12	650.36	1517.72	-0.0044	2	45
				765.39	1747.77			
				880.42	2223.01			
				1027.48				
				1155.58				
				1688.83				
				2282.10				
Manual annotation by matching observed MS/MS ion fragments with MS-Product Program								
DG2	N298	FMNYLLG[298]GSIPGTNDVLLAK	2254.19	930.52	393.36	N/A	N/A	N/A
				1027.65	1010.48			
				1284.82	1307.65			
				1398.76				
				1455.78				
DG2	N298	FMNYLLG[298]GSIPGTNDVLLAK	2238.23	543.38	1010.61	N/A	N/A	N/A
				658.41	1097.51			
				772.46				
				1027.63				
				1284.79				
				1398.76				

Supplementary references

11. Marie, B.; Zanella-Cléon, I.; Guichard, N.; Becchi, M.; Marin, F. Novel Proteins from the Calcifying Shell Matrix of the Pacific Oyster *Crassostrea gigas*. *Mar. Biotechnol.* **2011**, *13*, 1159–1168, doi:10.1007/s10126-011-9379-2.
40. Johnstone, M.B.; Wheeler, A.P.; Falwell, E.P.; Staton, M.E.; Saski, C.A.; Mount, A.S. Folian-Cv1 Is a Member of a Highly Acidic Phosphoprotein Class Derived From the Foliated Layer of the Eastern Oyster (*Crassostrea virginica*) Shell and Identified in Hemocytes and Mantle. *Front. Mar. Sci.* **2019**, *6*, 1–21, doi:10.3389/fmars.2019.00366.
44. Rivera-Pérez, C.; Hernández-Saavedra, N.Y. Review: Post-Translational Modifications of Marine Shell Matrix Proteins. *Comp. Biochem. Physiol. B. Biochem. Mol. Biol.* **2021**, *256*, 110641, doi:10.1016/j.cbpb.2021.110641.
46. Matsumura, K.; Nagano, M.; Fusetani, N. Purification of a Larval Settlement-Inducing Protein Complex (SIPC) of the Barnacle, *Balanus amphitrite*. *J. Exp. Zool.* **1998**, *281*, doi:10.1002/(SICI)1097-010X(19980501)281:1<12::AID-JEZ3>3.0.CO;2-F.
55. Miyashita, T.; Takagi, R.; Okushima, M.; Nakano, S.; Miyamoto, H.; Nishikawa, E.; Matsushiro, A. Complementary DNA Cloning and Characterization of Pearlin, a New Class of Matrix Protein in the Nacreous Layer of Oyster Pearls. *Mar. Biotechnol.* **2000**, *2*, 409–418, doi:10.1007/s101260000013.
56. Campbell, K.P.; MacLennan, D.H.; Jorgensen, A.O. Staining of the Ca2+-Binding Proteins, Calsequestrin, Calmodulin, Troponin C, and S-100, with the Cationic Carbocyanine Dye "Stains-All". *J. Biol. Chem.* **1983**, *258*, 11267–11273, doi:10.1016/S0021-9258(17)44413-9.
57. Mouchi, V.; Lartaud, F.; Guichard, N.; Immel, F.; de Rafélis, M.; Broussard, C.; Crowley, Q.G.; Marin, F. Chalky versus Foliated: A Discriminant Immunogold Labelling of Shell Microstructures in the Edible Oyster *Crassostrea gigas*. *Mar. Biol.* **2016**, *163*, 256, doi:10.1007/s00227-016-3040-6.
58. León, I.R.; da Costa Neves-Ferreira, A.G.; da Rocha, S.L.G.; de Oliveira Trugilho, M.R.; Perales, J.; Valente, R.H. Using Mass Spectrometry to Explore the Neglected Glycan Moieties of the Antiophidic Proteins DM43 and DM64. *Proteomics* **2012**, *12*, doi:10.1002/pmic.201200062.
60. Schmitt, P.; Gueguen, Y.; Desmarais, E.; Bachère, E.; de Lorgeril, J. Molecular Diversity of Antimicrobial Effectors in the Oyster *Crassostrea gigas*. *BMC Evol. Biol.* **2010**, *10*, 23, doi:10.1186/1471-2148-10-23.
79. Laemmli, U.K. Cleavage of Structural Proteins during the Assembly of the Head of Bacteriophage T4. *Nature* **1970**, *227*, 680–685.
83. Mann, K.; Edsinger-Gonzales, E.; Mann, M. In-Depth Proteomic Analysis of a Mollusc Shell: Acid-Soluble and Acid-Insoluble Matrix of the Limpet *Lottia gigantea*. *Proteome Sci.* **2012**, *10*, 28, doi:10.1186/1477-5956-10-28.
98. Goldberg, H.A.; Warner, K.J. The Staining of Acidic Proteins on Polyacrylamide Gels: Enhanced Sensitivity and Stability of "Stains-All" Staining in Combination with Silver Nitrate. *Anal. Biochem.* **1997**, *251*, 227–233, doi:10.1006/abio.1997.2252.
99. Samata, T.; Ikeda, D.; Kajikawa, A.; Sato, H.; Nogawa, C.; Yamada, D.; Yamazaki, R.; Akiyama, T. A Novel Phosphorylated Glycoprotein in the Shell Matrix of the Oyster *Crassostrea nippona*. *FEBS J.* **2008**, *275*, doi:10.1111/j.1742-4658.2008.06453.x.
100. Tarentino, A.L.; Gomez, C.M.; Plummer, T.H. Deglycosylation of Asparagine-Linked Glycans by Peptide:N-Glycosidase F. *Biochemistry* **1985**, *24*, 4665–4671, doi:10.1021/bi00338a028.
